# Lipid A analog CRX-527 conjugated to synthetic peptides enhances vaccination efficacy and tumor control

**DOI:** 10.1038/s41541-022-00484-y

**Published:** 2022-06-23

**Authors:** Elena Tondini, Niels R. M. Reintjens, Giulia Castello, Tsolere Arakelian, Marjolein Isendoorn, Marcel Camps, Jana Vree, Gijs A. van der Marel, Dmitri V. Filippov, Jeroen D. C. Codee, Ferry Ossendorp

**Affiliations:** 1 Department of Immunology, Leiden University Medical Center, Leiden University, Albinusdreef 2, 2333 ZA Leiden, The Netherlands; 2 Bio-organic Synthesis, Leiden Institute of Chemistry, Leiden University, Einsteinweg 55, 2333 CC Leiden, The Netherlands

**Keywords:** Conjugate vaccines, Adjuvants

## Abstract

Adjuvants play a determinant role in cancer vaccination by optimally activating APCs and shaping the T cell response. Bacterial-derived lipid A is one of the most potent immune-stimulators known, and is recognized via Toll-like receptor 4 (TLR4). In this study, we explore the use of the synthetic, non-toxic, lipid A analog CRX-527 as an adjuvant for peptide cancer vaccines. This well-defined adjuvant was covalently conjugated to antigenic peptides as a strategy to improve vaccine efficacy. We show that coupling of this TLR4 agonist to peptide antigens improves vaccine uptake by dendritic cells (DCs), maturation of DCs and T cell activation in vitro, and stimulates DC migration and functional T cell priming in vivo. This translates into enhanced tumor protection upon prophylactic and therapeutic vaccination via intradermal injection against B16-OVA melanoma and HPV-related TC1 tumors. These results highlight the potential of CRX-527 as an adjuvant for molecularly defined cancer vaccines, and support the design of adjuvant-peptide conjugates as a strategy to optimize vaccine formulation.

## Introduction

The success of immunotherapy in the treatment of cancer is largely dependent on the recruitment of tumor-specific T cells. T cells have the potential to recognize tumor exclusive antigens that are present on cancer cells and absent on healthy cells, such as overexpressed, viral or mutated proteins. Many pre-clinical and clinical studies have reported the potential of vaccination as a strategy to eradicate tumors, both by inducing de novo tumor-specific T cell responses or by reinforcing pre-existing ones^1^. However, it has also become apparent that vaccinating in the context of cancer is not trivial^2^. Tumors can develop several different evading mechanisms, such as the upregulation of inhibitory molecules, the recruitment of suppressor cells, and the induction of regulatory T cells^3^, causing T cell dysfunction and thereby hampering full therapeutic activity. To counteract these mechanisms, it is crucial to provide T cells with the appropriate signals during priming, in order to equip them with strong effector functions.

T cell priming and activation rely on vaccine uptake by properly activated dendritic cells (DCs), and research on enhancing vaccine efficacy has greatly been focused on optimizing the delivery of vaccine content to these cells. For example, delivery of nano-sized encapsulated vaccines, such as liposomes or nanoparticles, reduces the dispersal of vaccine components and promotes enhanced uptake by DCs, resulting in enhanced T cell responses and anti-tumor activity^4,5^. Alternatively, antigen and adjuvant are delivered to DCs via antibodies targeting DC molecules, such as DEC205 or DC-SIGN^6^. We and others have shown that the physical coupling of antigens and adjuvants, such as a Toll-like receptor (TLR) ligands, enables the delivery of maturation signals and antigens to the same dendritic cells, improving not only the numbers but also the quality of the generated T cell response^7–10^. Covalent attachment of Pam_3_CysSK_4_, a TLR2 ligand, to a peptide antigen greatly enhanced T cell induction as well as tumor control compared to the mixture of the two^11,12^. Peptide-based conjugates with an optimized Pam_3_CysSK_4_ analog are currently tested in the clinic for the treatment of HPV-associated malignancies^13^. Enhanced uptake, DC maturation and antigen presentation were also shown for conjugates bearing TLR7^14^, TLR9^15^, and NOD2 ligands^16,17^. We have recently disclosed the synthesis of a novel conjugate bearing a synthetic analog of the TLR4 ligand lipid A, CRX-527^18^. CRX-527 mimics lipid A^19^, the lipidic portion of bacterial lipopolysaccharides (LPS), maintaining its exceptional immune-stimulating activity while bypassing its toxicity, which had limited the use of lipid A as adjuvant in the clinic. In vivo priming of CD8 T cells by the CRX-527 conjugate induced differentiation of superior quality T cells compared to the mixture of the adjuvant and the peptide, reflecting the potential of this conjugate for cancer vaccination^18^.

We now show that peptide formulation as a lipid A conjugate improves antigen uptake and presentation by DCs in vitro, resulting in higher CD8 and CD4 T cell activation and cytokine production, and that prophylactic and therapeutic vaccination with CRX-527 conjugates containing tumor-specific cytotoxic T lymphocytes (CTL) or T-helper epitopes strongly promote T cell activation, resulting in effective tumor control in vivo.

## Results

### CRX-527 conjugates containing CTL or T-helper epitopes are immunologically active

**Fig. 1:**
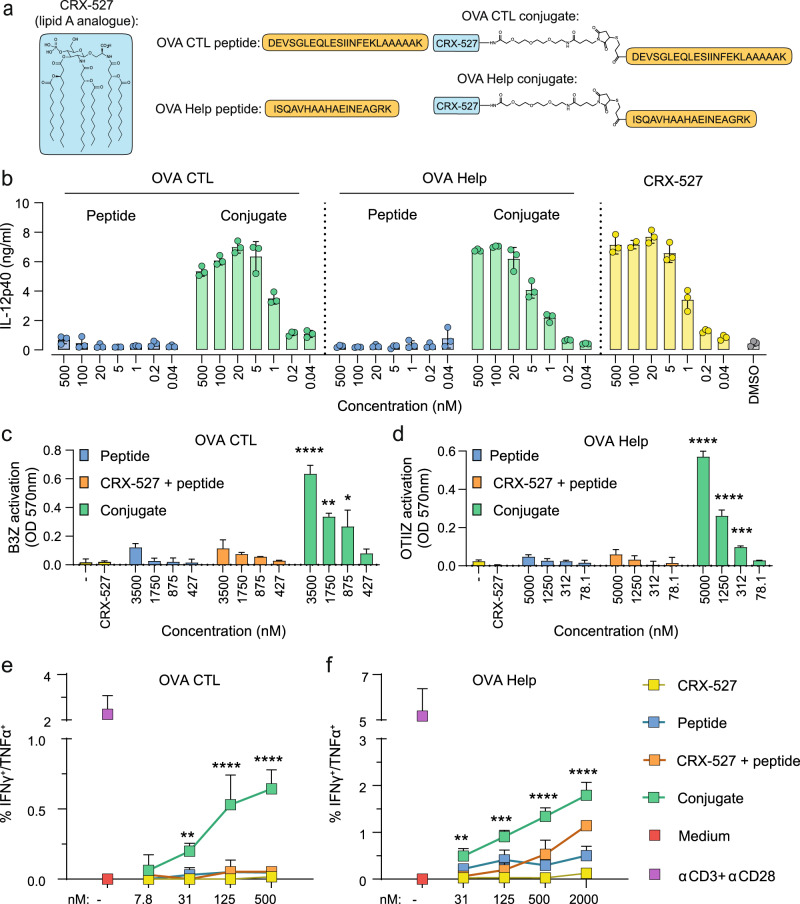
CRX-527-peptide conjugates are immunologically active and can be presented both on MHC class I and II complexes. **a** Schematic representation of the structures of the Lipid A analog CRX-527 and the OVA CTL and T-helper (OVA Help) peptide conjugates. **b** Concentration of IL-12p40 in the supernatant of D1 dendritic cells after overnight incubation with the indicated concentrations of peptides, conjugates, or CRX-527. **c** and **d** D1 DCs were pulsed for 2 h with the indicated concentration of compounds followed by overnight incubation with reporter hybridoma T cell lines. MHC class I presentation of the OVA CTL epitope was detected with the B3Z cell line (**c**); MHC class II presentation of the OVA Helper epitope was detected with the OTIIZ hybridoma (**d**) via colorimetric reaction. **e** and **f** D1 DCs were pulsed for 2 h with the indicated compounds at different concentrations and incubated for 48 h with purified naïve OT-I (**e**) or OT-II (**f**) TCR transgenic T cells. In the last 5 h, cells were incubated with Brefeldin A followed by staining for activation markers and cytokines. IFNγ/TNFα double producing OT-I (**e**) or OT-II (**f**) cells were detected by flow cytometry. Medium and stimulation with αCD3 + αCD28 antibodies were used as negative and positive controls, respectively. Statistical significance in all plots of conjugates versus mix was determined by two-way ANOVA followed by Dunnett’s multiple comparison test; *p < 0.05, **p < 0.01, ***p < 0.001, *****p < 0.0001. Experiments were performed in triplicates and are representative of two or three independent experiments with similar results, data are displayed as mean ± SD.

We previously reported a procedure to synthesize peptide conjugates bearing the lipid A analog CRX-527^18^. The ligand was equipped at the C6-position via an amide bond with a linker containing a maleimide and was conjugated to the N-terminus of a synthetic long peptide containing the model CTL epitope SIINFEKL via a thiol-ene coupling. This allowed proper internalization and class I presentation of the SIINFEKL epitope by DCs. Peptide loading of MHC class I and II molecules is dependent on different uptake and processing routes, therefore we tested whether conjugation of CRX-527 to a T-helper epitope would still allow for proper processing and epitope presentation in the MHC class II pathway. Two different long peptides containing either the CTL epitope or the T-helper epitopes derived from chicken ovalbumin were conjugated to the CRX-527 (Fig. 1a) and tested for their ability to induce DC maturation and antigen presentation in mouse dendritic cells. Both conjugates induced similar levels of DC maturation, as detected by IL-12 production (Fig. 1b), showing that the immune-stimulating properties of the lipid A analog are not affected by the conjugation to a long peptide. To evaluate antigen presentation, the conjugates were tested with the two hybridoma reporter T cell lines B3Z and OTIIZ, which possess TCR specificity for the CTL and the T-helper ovalbumin epitopes, respectively. These reporter T cell lines are not dependent on co-stimulation and their activation is therefore indicative of the actual levels of antigen presentation of the two epitopes. DCs were pulsed with the peptides, the conjugates, or an equimolar mixture of CRX-527 and peptides and incubated overnight with B3Z or OTIIZ cells. As shown in Fig. 1c, d, both CTL and T-helper conjugates strongly induce activation of the two hybridoma cell lines compared to free peptides. The mixture of CRX-527 and peptides only induced low T cell activation, indicating that conjugation enhances antigen presentation.

We next investigated the combined effect of antigen presentation and co-stimulation in vitro by incubating compound-pulsed DCs with purified naïve OT-I or OT-II T cells. After 48 h, T cells were analyzed for their proliferation, activation status and cytokine production. Although OT-I cells responded to DCs pulsed with the lipid A analog, antigen-specific proliferation was most efficient in presence of the CRX-527 conjugate (Supplementary Fig. 1a). This was associated with upregulation of the early activation marker CD69 and the activation marker and IL-2 receptor CD25 (Supplementary Fig. 1a). In addition, conjugate-activated OT-I cells produced high levels of IFNγ and IL-2 as detected in culture supernatants (Supplementary Fig. 1b). The ability to produce multiple cytokines is a measure of T cell quality^20,21^. Cells were analyzed for polyfunctionality of cytokine responses by intracellular cytokine staining. While DCs pulsed with peptide alone or in combination with soluble CRX-527 elicited detectable production of IFNγ and TNFα in <3% of OT-I cells (Supplementary Fig. 1c), the CTL conjugate could stimulate up to 30% of total OT-I. The majority of these cells were single IFNγ-producers; however, a small but significant percentage was positive for both IFNγ and TNFα, indicating the potency of CTL conjugates to elicit polyfunctional responses in vitro (Fig. 1e).

In contrast to OT-I, OT-II cells displayed antigen-specific proliferation irrespective of CRX-527 conjugation (Supplementary Fig. 1d). Antigen-dependent stimulation induced expression of CD25 and CD69 activation markers (Supplementary Fig. 1d), as well as IFNγ and IL-2 production (Supplementary Fig. 1e). IFNγ production was significantly higher in the presence of CRX-527, both in the conjugate and in the mixture. Analysis of cytokine polyfunctionality by intracellular staining displayed a majority of IFNγ and TNFα single producers (Supplementary Fig. 1f). Also here, the T-helper conjugate induced the highest percentage of responding cells, reaching around 15% of total cells. Moreover, OT-II cells primed with the CRX-527-conjugated peptide displayed a significant increase in IFNγ^+^/TNFα^+^ double producing OT-II cells (Fig. 1f).

OT-II cells generally show a relatively weak functional response compared to OT-I. To extend these findings to another CD4 T helper cell model, we synthesized a conjugate with a peptide containing the CD4 epitope (EnvH) derived from the Env protein of Murine Leukemia virus (Supplementary Fig. 2a)^22^. The EnvH conjugate displayed similar activity to the OVA Help conjugate in terms of DC maturation (Supplementary Fig. 2b). We evaluated T cell activation by the conjugate via two different T cell models. First, we analyzed antigen presentation of conjugates to the hybridoma cell line 3A12 derived from a EnvH-specific T cell clone, which expresses the reporter enzyme beta-galactosidase upon TCR triggering. DCs pulsed with the EnvH conjugate induced significantly stronger 3A12 activation than peptide alone or mixed with CRX-527 (Supplementary Fig. 2c). Secondly, we co-cultured pulsed DCs with purified CD4 T cells isolated from the TCR transgenic mouse MolH, which are specific for the envH epitope. Similarly to OT-II, the EnvH conjugate induced significant TNFα production in MolH T cells compared to its unconjugated counterparts (Supplementary Fig. 2d). Therefore, the EnvH conjugate was also able to enhance CD4 T cell activation.

To summarize, we demonstrated for three different peptides that conjugation of the lipid A analog CRX-527 retains the immunological properties of the two components, preserving both ligand activity as well as epitope presentation on MHC class I and II molecules. In addition, conjugation improves antigen uptake and processing by DCs as well as CD8 and CD4 T cell activation of naïve T cells in vitro, as indicated by enhanced upregulation of activation markers and polyfunctional cytokine profile.

### CRX-527 conjugated to CTL or T-helper peptides impact dendritic cell activation and T cell priming in vivo

**Fig. 2:**
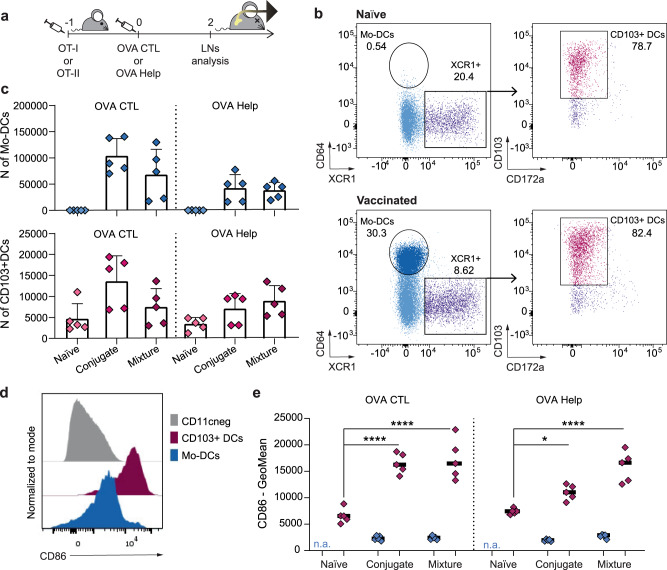
OVA CTL and Help peptide conjugated to CRX-527 promote DCs influx in the draining lymph node upon in vivo injection. **a** Mice (*n* = 5 per group) were adoptively transferred with CFSE labeled OT-I or OT-II cells 24 h before intradermally receiving 2 nmol of OVA CTL or Help CRX-527 conjugates, or an equimolar mix of peptide and CRX-527. 48 h later the inguinal lymph nodes were harvested for analysis. **b** Representative dot plots for the gating of Mo-DCs and CD103+ dermal DCs in naïve versus vaccinated mice. Cells were pre-gated to exclude dead/CD19^neg^/CD11c^neg^/MHC class II^neg^ cells (**c**). Absolute count of Mo-DCs and XCR1+/CD103+ DCs in the inguinal lymph nodes (**d**). Representative histograms of CD86 expression in non-APCs (CD11c^neg^), Mo-DCs or XCR1+/CD103+ DCs. **e** Fluorescence intensity of CD86 signal (GeoMean) in Mo-DCs and XCR1+/CD103+ DCs subsets upon vaccination. Statistical significance was determined by one-way ANOVA followed by Tukey’s multiple comparison test; *p < 0.05, *****p < 0.0001; data are displayed as mean ± SD.

**Fig. 3:**
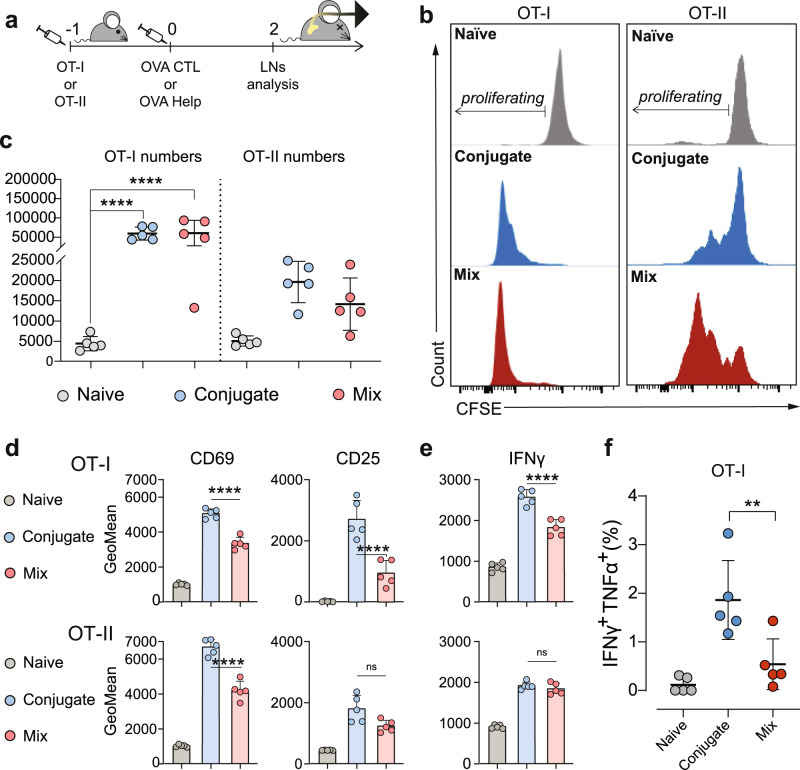
OVA CTL and Help Lipid A-peptide conjugates promote enhanced activation of T cells upon in vivo injection. **a** Mice (*n* = 5 per group) were adoptively transferred with CFSE labeled OT-I or OT-II cells 24 h before intradermally receiving 2 nmol of OVA CTL or Help CRX-527conjugates, or an equimolar mix of peptide and CRX-527. 48 h later, the inguinal lymph nodes were harvested for analysis of OT-I or OT-II T cell proliferation and activation. **b** Representative histograms of CFSE signal in labeled OT-I or OT-II cells. **c** Absolute count of total OT-I and OT-II cells in pooled inguinal lymph nodes. **d** Mean fluorescence intensity (GeoMean) of CD69 and CD25 T cell activation markers in OT-I (upper) or OT-II (lower) cells as detected by flow cytometry. **e** Mean fluorescence intensity of IFNγ cytokine in OT-I (upper) or OT-II (lower) as detected by flow cytometry. Statistical significance of the conjugates compared to the mix in both (**d**) and (**e**) was determined by one-way ANOVA followed by Sidak’s multiple comparison test; *****p < 0.0001. **f** Percentage of IFNγ/TNFα double producing OT-I cells. Statistical significance was determined by one-way ANOVA followed by Tukey’s multiple comparison test; **p < 0.01, all data are displayed as mean ± SD.

The effect on T cell activation of the conjugated vaccines was subsequently assessed in vivo (Fig. 2a). The two formulations were injected intradermally in mice which were previously adoptively transferred with CFSE-labeled OT-I or OT-II T cells. Two days after vaccination, T cells were analyzed in the inguinal lymph nodes (LNs) draining the vaccination site (Fig. 3a). In the LNs of vaccinated mice, the total cell number significantly increased compared to naïve mice (Supplementary Fig. S3a and S3b). This coincided with high numbers of CD11c^+^/MHC-II^+^ antigen-presenting cells (Supplementary Fig. 3c) in the same lymph nodes, indicating that the CRX-527 conjugates are immunologically active and capable of mobilizing innate immune cells. We detected a significant increase of monocyte-derived dendritic cells (Mo-DCs, Fig. 2b, c), which are known to be strongly induced upon TLR4 stimulation^23^. Moreover, albeit in lower numbers, we observed a strong influx of XCR1^+^/CD103^+^ migratory dermal DCs, the key DC subset involved in the transport and presentation of peripheral antigens and T cell priming^24^. In addition, both DC subsets showed expression of the co-stimulatory molecule CD86 (Fig. 2d, e). Correlating with the intradermal site of injection of the lipid A analog, the migratory dermal DCs displayed significantly higher CD86 expression than Mo-DCs (Fig. 2e).

OT-I and OT-II T cell numbers in the LNs were also strongly increased in vaccinated mice. All OT-I cells underwent complete proliferation in both vaccination groups, while a portion of OT-II cells still remained undivided (Fig. 3b). The total numbers of OT-I and OT-II in the LNs were higher than in unvaccinated mice (Fig. 3c). Analysis of activation markers in OT-I CD8 T cells revealed increased expression of all markers analyzed (CD69, CD25, ICOS) in mice receiving the conjugated CTL vaccine in comparison to the mixture of CRX-527 and peptide (Fig. 3d upper panel and Supplementary Fig. 3d). Increased CD69 upregulation was also observed in proliferated OT-II CD4 T cells in the T-helper conjugate group (Fig. 3d, lower panel), while there was no detectable difference between T-helper conjugate or mixture groups in the other analyzed markers (Fig. 3d lower panel and S3d). Analysis of intracellular IFNγ and TNFα cytokine production showed that significantly higher levels of IFNγ were produced by OT-I T cells in mice vaccinated with the conjugate (Fig. 3e upper panel). This group also displayed a higher percentage of IFNγ/TNFα double producing T cells (Fig. 3f). IFNγ production was also detected in proliferating OT-II cells, and similar levels were induced by conjugated T-helper vaccine or mixed vaccine (Fig. 3e, lower panel). No detectable TNFα production was observed in OT-II.

In summary, these data show that conjugated CTL and T-helper peptide vaccines induce enhanced T cell activation and potent expansion upon priming in vivo. Similar to what observed in vitro, this effect was more pronounced in CD8 T cells than in CD4 T cells.

### Prophylactic vaccination with CRX-527 conjugates improves tumor control against B16OVA melanoma

**Fig. 4:**
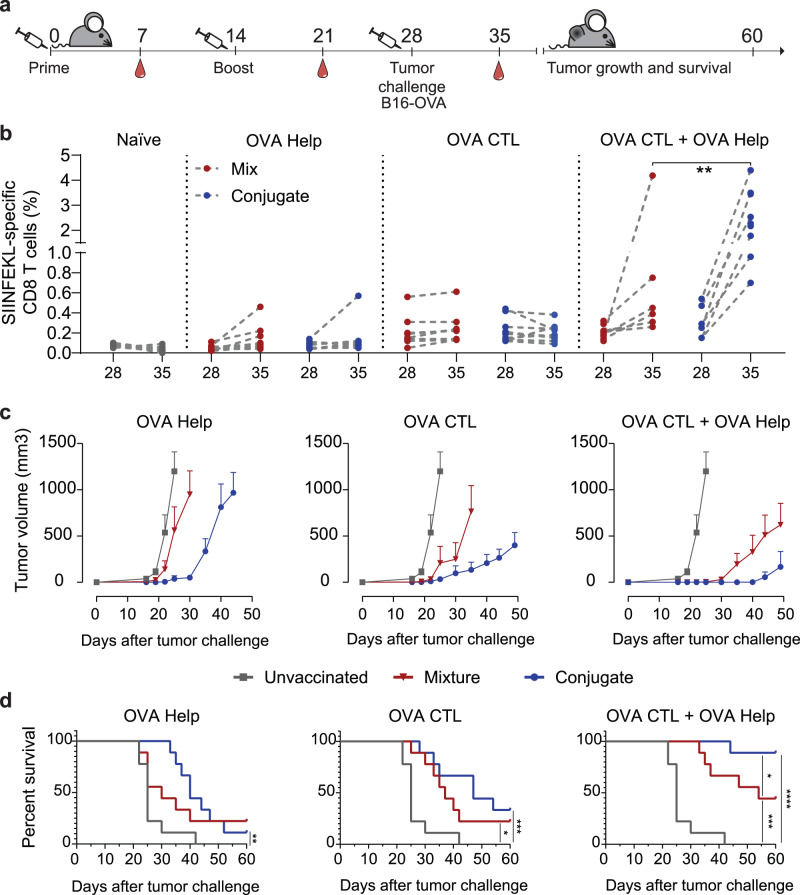
Prophylactic vaccination with CRX-527-peptide conjugates potentiates tumor protection compared to unconjugated vaccines. **a** Schematic overview of the experiment. Mice (*n* = 9) were primed and boosted at an interval of 2 weeks and later challenged with 100,000 B16OVA tumor cells. At the indicated time points, blood was withdrawn to analyze SIINFEKL-specific CD8 responses by tetramer staining. **b** Frequencies of SIINFEKL-specific CD8 T cell responses in all vaccinated and unvaccinated groups on the day of B16-OVA challenge and 7 days later. **c** Average B16-OVA tumor growth in groups that were unvaccinated (gray line) or vaccinated with either CRX-527 conjugate (blue line) or a mixture of CRX-527 and peptide (red line) containing OVA CTL or Helper epitopes. Data are displayed as mean ± SD. **d** Overall survival of prophylactically vaccinated mice with the indicated epitopes in form of CRX-527 conjugates or mixture. Statistical significance was determined by Log-rank Mantel–Cox test; *p < 0.05, **p < 0.01, ***p < 0.001, *****p < 0.0001.

We next evaluated whether vaccination efficacy of conjugate vaccines translated into improved endogenous T cell effector responses against a lethal challenge with B16OVA melanoma cells (Fig. 4a). Mice were primed and boosted in an interval of 2 weeks with the conjugates or, as control groups, a mixture of the lipid A analog and the OVA CTL and T-helper peptides, separately or in combination. The induction of SIINFEKL-specific responses in the groups that received CTL peptide was measured in blood 7 days after each vaccine injection by H-2K^b^-SIINFEKL tetramer staining (Supplementary Fig. 4a). The levels of SIINFEKL-specific responses were similar for conjugates and mixture in the groups that received the CTL peptide only. In the groups in which the T-helper epitope was included, the levels of the OVA-specific CD8 responses were significantly increased, underlining the critical role that CD4 T cell help plays during the priming of CTL responses. After boost, a significantly higher frequency of SIINFEKL-specific CD8 T cells was observed in mice vaccinated with both CTL and T-helper CRX-527 conjugates (Supplementary Fig. 4a).

The level of SIINFEKL-specific T cells in response to B16OVA challenge was measured in all vaccinated groups on the day of tumor inoculation and 7 days later. Naïve and T-helper-vaccinated mice did not display any SIINFEKL-specific responses on the day of tumor challenge; however, some CD8 responses appeared one week later in some of the mice that were vaccinated with the T-helper conjugate or the mixture (Fig. 4b, left panels). These responses were not detected in unvaccinated mice and can be attributed to the presence of tumor-specific T-helper responses, which facilitated tumor-induced priming of SIINFEKL-specific responses. In the groups that were vaccinated with the CTL epitope, the levels of SIINFEKL-specific responses were detectable on the day of tumor challenge and one week after. The groups that received only CTL peptide did not show any tumor-specific expansion or decrease of the SIINFEKL-specific response (Fig. 4b, middle panel). In contrast, the groups vaccinated with the combination of the CTL and T-helper peptides displayed a significant expansion of SIINFEKL responses after tumor injection. Notably, the mice that received the conjugated vaccine displayed the highest frequencies (up to 5%) of total CD8 T cells of all groups (Fig. 4b, right panel). The ability of these responses to immunologically protect mice from tumor growth was evaluated by following tumor sizes over 60 days (Fig. 4c). All unvaccinated mice developed a tumor within 20 days. Mice vaccinated with the CRX-527-T-helper conjugated or unconjugated vaccine experienced a short delay in tumor growth (Fig. 4c, left panel) which was more pronounced in the mice that received the conjugate vaccine and resulted in a significant increase of survival (Fig. 4d). The groups that were vaccinated with the CTL epitope displayed a more accentuated delay in tumor growth, which was stronger in the mice vaccinated with the CRX-527 conjugate compared to the mixture (Fig. 4c, middle panel). Finally, the groups that were vaccinated with both peptides showed the most effective protection from tumor growth (Fig. 4c, right panel). The mixture of the lipid A analog and peptides protected less efficiently than the conjugated peptides, which resulted in long-term overall survival of about 90% of animals (Fig. 4d).

These results highlight superior quality of the T cells induced by the CRX-527 conjugates, which translates into superior control of aggressive tumor growth, and underline the importance of combining CD4 and CD8 conjugate vaccines for optimal induction of functional T cell responses.

### Therapeutic efficacy of OVA CTL and T-helper conjugates against B16OVA and TC1 tumors

**Fig. 5:**
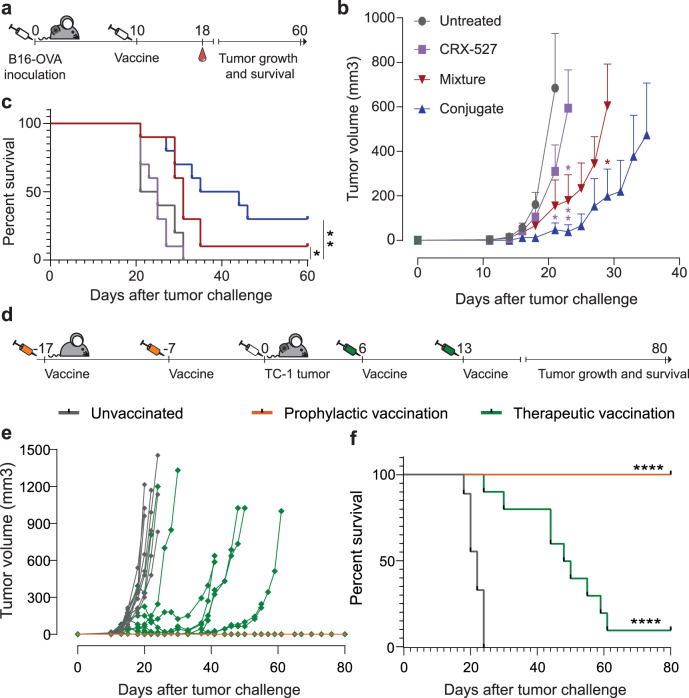
Therapeutic vaccination with CRX-527 conjugates results in effective therapeutic activity. **a** Schematic overview of the experiment. Mice (*n* = 10 per group) were injected with 100,000 B16OVA tumor cells. Once tumors were palpable, mice were vaccinated with 1 nmol of CRX-527, a mixture of CRX-527 and OVA CTL and Help peptides or the two OVA CTL and Help conjugates. Blood was withdrawn at day 18 to monitor the induction of SIINFEKL-specific CD8 responses by tetramer staining. **b** Average (±SD) B16OVA tumor growth in the different therapeutic vaccination groups. Statistical significance between Lipid A, mix and conjugates at different time points was calculated by one-way ANOVA and Tukey’s multiple comparison. The color of the stars indicates to which group the statistical significance is referred to. *p < 0.05, **p < 0.01. **c** Overall survival of mice therapeutically vaccinated with CRX-527or OVA CTL and Help peptides in form of mix with CRX-527 or conjugates. Statistical significance was determined by Log-rank Mantel–Cox test; *p < 0.05, ** *p* < 0.01. **d** Schematic representation of prophylactic or therapeutic vaccination experiment with HPV E7 specific CRX-527 conjugates against TC-1 tumor. Mice (*n* = 10 per group) were vaccinated either before (orange syringes) or after (green syringes) TC-1 tumor inoculation and tumor growth and survival were monitored for 80 days. **e** Individual tumor growth of unvaccinated (gray), prophylactically vaccinated (orange) and therapeutically vaccinated (green) mice and **f** their corresponding survival plot. Statistical significance was determined by Log-rank Mantel–Cox test; ****p < 0.0001.

Therapeutic efficacy of the CRX-527-conjugated vaccines was evaluated in mice with established B16OVA melanoma tumors that were intradermally injected with a combination of the OVA CTL and T-helper peptides 10 days after tumor inoculation. The conjugate vaccine was compared to free CRX-527 and an equimolar mix of the OVA peptides with lipid A analog. Tumor growth was significantly delayed by the therapeutic vaccinations (Fig. 5a) but not by CRX-527 alone. In particular, the conjugated vaccine exhibited the highest therapeutic effect on overall survival rate (Fig. 5b, c). The frequency in blood of the induced SIINFEKL-CD8 T cell responses did not detectably differ between groups (Supplementary Fig. 4b), however tumor immune control by the conjugated vaccine was significantly more efficient.

Finally, we analyzed whether the conjugation of the lipid A analog could be applied to another antigenic peptide cancer model. CRX-527 was conjugated to a synthetic long peptide containing the CTL epitope, derived from the E7 protein of the Human Papilloma Virus (HPV) type 16. Mice were vaccinated either prophylactically or therapeutically against TC-1 tumors expressing the HPV E7 protein. Prophylactic vaccination protected all animals long term from tumor challenge, while therapeutic vaccination could cure 10% of the animals, and delay tumor growth in 80% of mice (Fig. 5e), resulting in significantly increased overall survival rate (Fig. 5f). Overall, these data demonstrate the potential of CRX-527-peptide conjugates in improving tumor antigen-specific T cell responses for effective cancer vaccination.

## Discussion

In this study, we report the efficacy of self-adjuvanting peptide vaccines containing the potent TLR4 ligand lipid A analog CRX-527 in mediating tumor control. Therapeutic cancer vaccination is a promising specific immunotherapeutic approach but presents itself with the challenge of inducing functional T cells in the context of a suppressed immune environment. For this reason, it is crucial to design vaccines that maximize T cell priming conditions. T cell priming is a multifactorial process that can be modulated at different levels. To start, vaccine efficacy strongly depends on its ability to reach dendritic cells. We show that conjugation of peptide antigen to CRX-527 enhances vaccine uptake and antigen presentation by DCs to T cells in vitro. Direct conjugation of antigen to pathogen-associated molecular patterns (PAMPs) as a strategy to improve DC targeting and uptake has been described for multiple ligands, including TLR ligands^15,25,26^. TLRs are not necessarily involved in the antigen uptake process^15^, however DCs express multiple scavenger and uptake receptors that mediate constant antigen uptake and that can also present affinity for PAMPs^27^. Notably, TLR signaling from the endosomes plays a role in phagosome maturation and in directing the cargo towards antigen processing and MHC-loading routes, rather than the degradation compartments. This has also been described for TLR4 and it has been reported that engagement of TLR4 within the antigen-containing endosomes causes delay in antigen degradation and enhanced MHC-I cross-presentation^28–30^ as well as MHC-II presentation^31,32^.

In vivo, antigen uptake and presentation can be mediated by different DCs subsets. Upon intradermal injection, the vaccine needs to reach and mobilize dermal DCs, which will migrate to the LNs to induce T cell priming. We observed an influx of XCR1^+^/CD103^+^ DCs. Among the different DC types, this subset of migratory dermal DCs plays a critical role in antigen transport from the periphery^24,33^ and is involved in antigen presentation and T cell priming. Moreover, migratory dermal DCs have a recognized role in antigen transport from the tumor site^34^ and affect therapeutic efficacy of checkpoint immunotherapies^35,36^. We observed increased influx of these DCs and upregulation of CD86. In addition, we detected a strong influx of CD64^+^ Mo-DCs. TLR4 stimulation has been described to strongly induce recruitment of monocytes and differentiation into Mo-DCs^23,37^. This cell subset supports inflammation and immune activation as well as antigen presentation^38,39^. It remains to be determined, however, how these two subsets contribute to immune activation and antigen presentation in our system.

The nature of the adjuvants influences the shaping of the response during T cell priming. LPS toxicity has long prevented the clinical exploitation of the TLR4 pathway. However, the emergence of novel detoxified ligands has recently made targeting of TLR4 a successful strategy with various applications. Monophosphoryl lipid A (MPL), a member of the LPS family isolated from *Salmonella*, is a component of commercially available prophylactic vaccines against human papilloma, hepatitis B, herpes zoster viruses and malaria. This adjuvant enables skewing of T cell responses towards Th1 immunity^40^. More recently GLA-SE, a similar lipid A analog in a stable emulsion, has entered clinical evaluation as vaccine adjuvants for H5N1 influenza^41^ as well as tuberculosis^42^. This adjuvant also displays noticeable activity for cancer immunotherapy. In a pre-clinical setting, intra-tumoral injection of GLA-SE was described to synergize with vaccination or adoptive T cell transfer to mediate complete tumor regression^43^. This adjuvant is currently being tested in the clinic in booster vaccination against the NY-ESO cancer testis antigen after prime with an adenoviral vector^44^, in melanoma^45^ and as intra-tumoral monotherapy^46^. Here, we report the first-time employment of CRX-527 as adjuvant in defined self-adjuvanting cancer vaccines. This molecularly well-defined adjuvant retains strong TLR4 activating properties, is more potent than MPL^18^, and was well tolerated upon injection. Most importantly, it shows strong efficacy at nanomolar doses, induction of Th1 skewed immunity and excellent pre-clinical activity in tumor control.

Finally, priming efficacy is also influenced by the presence of CD4 T-helper cells. By analyzing self-adjuvanting vaccines containing either a CTL or a T-helper epitope separately or together, we observe maximal tumor protection when these two epitopes are combined. CTLs primed in concomitance with CD4 T cell help provided by the T-helper epitope display increased expansion potential and the highest ability of protecting from tumor growth. The presence of Helper T cells during priming strongly stimulates the breadth of the response induced and determines cytotoxic T cell effector function^47^. Absence of T cell help during priming can result in sub-optimally primed CTL responses with phenotype resembling exhausted T cells^48^. Tumors are particularly effective in modulating T cells to exhaustion and it is therefore essential to include CD4 T cell epitopes in the design of an effective cancer vaccine, preferably from a protein that is also expressed by the tumor cells to favor help at the local level^22,49^.

In conclusion, we have developed a versatile, molecularly well-defined, peptide-based vaccine system which incorporates the potent TLR4 ligand, CRX-527, in peptide-adjuvant conjugates for cancer vaccination. Physical conjugation of this adjuvant to different peptides potentiates dendritic cell functions, effector T cell activation and expansion, which translates into effective anti-tumor immunity in vivo, representing a promising platform for specific immunotherapy in the clinic.

## Methods

### Cell culture

The D1 cell line is a growth factor-dependent spleen-derived immature DC cell line from C57BL/6 (H-2^b^) mice. D1 cells were cultured in IMDM medium supplemented with GM-CSF supernatant^50^. The B3Z, OTIIZ, and 3A12 hybridoma cell lines were cultured in IMDM medium (Lonza, Basel, Switzerland) supplemented with 8% FCS (Greiner, Kremsmünster, Austria), penicillin and streptomycin, glutamine (Gibco, Carlsbad, CA, USA), β-mercaptoethanol (Merck, Kenilworth, NJ, USA), and hygromycin B (AG Scientific Inc., San Diego, CA, USA) to maintain expression of the beta-galactosidase reporter gene. The B16OVA and TC-1 tumor cell lines were cultured in IMDM medium (Lonza, Basel, Switzerland) supplemented with 8% FCS (Greiner, Kremsmünster, Austria), non-essential amino acids, sodium pyruvate, glutamine, penicillin and streptomycin, (all from Gibco, Carlsbad, CA, USA), β-mercaptoethanol (Merck, Kenilworth, NJ, USA). G418 (Life Technologies, Carlsbad, CA, USA) was used to maintain OVA expression in B16OVA cells and E6 and E7 expression in TC-1.

### Synthesis of peptides and CRX-527 peptide conjugates

The peptides used in this study were: the ovalbumin peptide OVA_248-265_ DEVSGLEQLESIINFEKLAAAAAK and OVA_323-341_ ISQAVHAAHAEINEAGRK, the peptide GQAEDRAHYNIVTFBBKBDSTLRLBVK, containing the CTL epitope from the E7_42–70_ protein of the Human Papilloma Virus type 16, and the helper peptide EEPLTSLTPRCNTAWNRL derived from the envelop_118–135_ protein of the Moloney Murine Leukemia virus. All four peptides were synthesized using automated peptide synthesis and purified via reversed-phase high-performance liquid chromatography (RP-HPLC). For conjugation with CRX-527, the peptides were assembled with 3‐(tritylthio)propionic acid at the N-terminus prior cleaving from the resin. The CRX-527 peptide conjugates were generated and purified using the described methods in ref. ^18^. The CRX-527 HPV-conjugate was purified via HPLC chromatography. More detailed experimental procedures for conjugate synthesis and LCMS and MALDI spectra can be found in Supplementary Information.

### Animals

For vaccination and tumor experiments, 6–8 weeks old female C57BL/6 were purchased from Charles River Laboratories. The TCR transgenic OT-I and OT-II mouse strains were obtained from Jackson Laboratory and maintained on CD45.1^+^ C57BL/6 background.

The T-cell receptor (TCR) transgenic mouse strain MolH was generated on a C57BL/6 background at the Leiden University Medical Center (LUMC). MolH mice express a TCRαβ recognizing the Moloney MuLV envH_119–137_ peptide presented by MHC class II I-A^b^. Mice were housed in specific pathogen-free (SPF) conditions at the LUMC animal facility. All animal experimentations were approved by and according to guidelines of the Dutch Animal Ethical Committee.

### In vitro DC maturation assay

The test compounds were dissolved in DMSO at a concentration of 500 µM and sonicated in water bath for 15 min. 50,000 D1 cells were seeded in triplicates in 96-well round bottom plates (Corning, Amsterdam, The Netherlands) in 100 µl medium. Two times concentrated compounds were titrated in medium and 100 µl were added on top of D1. After 24 h of incubation at 37 °C, supernatant was taken from the wells to measure the amount of produced IL-12p40 by ELISA assay (BioLegend, San Diego, CA, USA) according to manufacturer instructions.

### In vitro antigen presentation assay

The test compounds were dissolved in DMSO at a concentration of 500 µM and sonicated in water bath for 15 min. 50,000 D1 cells were seeded in triplicates in 96-well flat bottom plates (Corning, Amsterdam, The Netherlands) and pulsed for 2 h with 200 µl of the test compounds in medium at the indicated concentrations. After 2 h, cells were washed once with 200 µl of fresh medium. For B3Z and OTIIZ assay, 50,000 B3Z or OTIIZ cells were added per well in 200 µl of medium and incubated with D1 cells overnight. The following day TCR activation was detected by measurement of absorbance at 595 nm upon color conversion of chlorophenol red-β-d-galactopyranoside (Calbiochem®, Merck, Bullington, MA, USA) by the beta-galactosidase enzyme. For OT-I and OT-II T cell stimulations, CD8 and CD4 T cells were isolated from the spleens and lymph nodes of naïve OT-I and OT-II transgenic mice, respectively, by using CD8 or CD4 negative selection kits (BD Biosciences, San Jose, CA, USA) according to manufacturer instructions. The cells were labeled with 5 µM CFSE (Invitrogen, Carlsbad, CA, USA) at 37 °C for 10 min and 50,000 cells per well were added on top of D1 cells. After 36 h, supernatant was collected for the detection of INFγ and IL-2 production (BioLegend, San Diego, CA, USA) and brefeldin A (Sigma-Aldrich, St. Louis, MO, USA) at 5 µg/ml was added for 6 h. At the end of the incubation, the cells were stained for FACS analysis and acquired on BD FACS LSR II 4L Full (BD Biosciences, San Jose, CA, USA).

### In vivo antigen presentation

CD8 and CD4 T cells were isolated from the spleens and lymph nodes of naïve OT-I, OT-II, or MolH transgenic mice, respectively, by using CD8 or CD4 negative selection kits (BD Biosciences, San Jose, CA, USA) according to manufacturer instructions. The cells were labeled with 5 µM CFSE (Invitrogen, Carlsbad, CA, USA) at 37 °C for 10 min and 1,000,000 OT-I or OT-II cells were injected intravenously in naïve C57BL/6 mice. On the next day, mice received an intradermal injection of 2 nmol of either OVA CTL peptide + CRX-527 conjugated or mixed, OVA T-helper peptide + CRX-527 conjugated or mixed, or vehicle (saline solution). To prepare the vaccine. the different compounds were dissolved in DMSO at a concentration of 500 µM and sonicated in water bath for 15 min. The required amounts for vaccination were mixed to saline solution and 30 µl per mouse were injected. After 48 h, the inguinal lymph nodes were harvested and single cells suspension were obtained. A portion of these cells was used for direct staining for either DC or T cell analysis, while a portion was incubated with 5 µg/ml of Brefeldin A (Sigma-Aldrich, St. Louis, MO, USA) for 6 h and was subsequently stained for cytokines. Precision count beads (Biolegend, San Diego, CA, USA) were added in some samples to allow cell quantification. Samples were acquired on BD FACS LSR II 4L Full (BD Biosciences, San Jose, CA, USA).

### Prophylactic vaccination and B16-OVA tumor challenge

Naïve 6–8 weeks old C57BL/6 female mice were injected intradermally at the tail base with 2 nmol of the indicated conjugates or an equimolar mix of CRX-527 and peptide. To prepare the vaccine, the different compounds were dissolved in DMSO at a concentration of 500 µM and sonicated in water bath for 15 min. The required amounts for vaccination were added to saline solution and 30 µl per mouse were injected. Fourteen days later, the animals were boosted with the same vaccine formulations. After 28 days, 50,000 B16-OVA cells were injected subcutaneously in the flank and tumor growth was monitored. At different time points during the experiments, 20 µl of blood were collected from the tail vein for detection of SIINFEKL-specific T cell responses via SIINFEKL-H-2K^b^ tetramer staining. Mice were sacrificed when the tumor volume surpassed 1500 mm^3^.

### Therapeutic vaccination against B16-OVA tumors

Naïve C57BL/6 female mice were injected subcutaneously in the flank with 50,000 B16OVA cells and tumor growth was monitored. When tumors reached a palpable size with an estimated volume of around 1 mm^3^ (day 10), mice were vaccinated with 2 nmol of CRX-527, conjugated OVA peptides, or a mix of CRX-527 and OVA peptides. Eight days later, 20 µl of blood were collected from the tail vein for the detection of SIINFEKL-specific T cell responses via SIINFEKL-H-2K^b^ tetramer staining. Tumor growth was monitored over time and mice were sacrificed when the tumor volume surpassed 1000 mm^3^ in conformance to ethical regulations for animal welfare.

### TC-1 tumor challenge

Mice were subcutaneously injected in the flank with 100,000 TC-1 cells. For prophylactic vaccination, mice were vaccinated with 2 nmol of E7 peptide conjugated to CRX-527, or an equimolar mixture of the two, 17 and 7 days before tumor challenge. For therapeutic vaccination, mice were vaccinated with 2 nmol of compounds 6 and 13 days after tumor challenge. Tumor growth was monitored over time and mice were sacrificed when the tumor volume surpassed 1000 mm^3^ in conformance to ethical regulations for animal welfare.

### Flow cytometry staining and antibodies

For flow cytometry staining, cells were washed and stained in PBA buffer (0.5% BSA, 0.02% sodium azide in PBS) for 30 min on ice. For intracellular cytokine staining, cells were fixed and permeabilised with Intracellular Staining Permeabilization Wash Buffer (Biolegend, San Diego, CA, USA). Antibodies used were: PE-Cy7 anti-CD86 (Cat. No. 560582, 1:100), PE-Cy7 anti-IFNγ (Cat. No. 557649, 1:200), both from BD Pharmigen, San Diego, CA, USA), APC-R700 anti-CD8 (Cat. No. 564983, 1:500), V500 anti-I-A/I-E (Cat. No. 562366, 1:1000, all from BD Horizon, Franklin Lakes, NJ, USA), PE-Dazzle 594 anti-TNFα (Cat. No. 506345, 1:100), PerCP-Cy5.5 anti-CD8a (Cat. No. 100733, 1:500), PerCP-Cy5.5 anti-CD172a (Cat. No. 144009, 1:100), BV711 ant-CD25 (Cat. No. 10204, 1:200), BV605 anti-Ly6C (Cat. No. 128035, 1:1000), BV786 anti-XCR1 (Cat. No. 148225, 1:100), BV711 anti-CD64 (Cat. No. 139311, 1:100), BV650 anti-CD19 (Cat. No. 115541, 1:100), Alexa Fluor 700 anti-CD45.1 (Cat. No. 110723, 1:100, all from Biolegend, San Diego, CA, USA), eF450 anti-CD45.1 (Cat. No. 48-0453-82, 1:100), PE anti-CD103 (Cat. No. 12-1031-82, 1:200), Alexa Fluor 700 anti-CD4 (Cat. No. 56-0041-82, 1:200), Fixable Viability Dye eFluor780 (Cat. No. 65-0865-14, 1:1000 all from eBioscience, San Diego, CA, USA). APC or PE labeled H-2K^b^-SIINFEKL tetramers were made in-house and used at dilution 1:200.

### Statistical analysis

Results are expressed as mean ± SD. Statistical significance among groups was determined by multiple comparison using the GraphPad software after ANOVA and multiple comparison test. Details of the used tests are described in the legends. Cumulative survival time was calculated by the Kaplan–Meier method, and the log-rank test was applied to compare survival between two groups. *P*-values of ≤0.05 were considered statistically significant.

### Reporting summary

Further information on research design is available in the Nature Research Reporting Summary linked to this article.

## Supplementary information

Supplementary Material

REPORTING SUMMARY

## Data Availability

The data that support the findings of this study are available from the corresponding author upon reasonable request.
