# Supplementary material for: Lipid A analog CRX-527 conjugated to synthetic peptides enhances vaccination efficacy and tumor control
Source: NPJ Vaccines. 2022 Jun 23;7:64. doi: 10.1038/s41541-022-00484-y (PMC9226002; doi:10.1038/s41541-022-00484-y)

Figure S1:

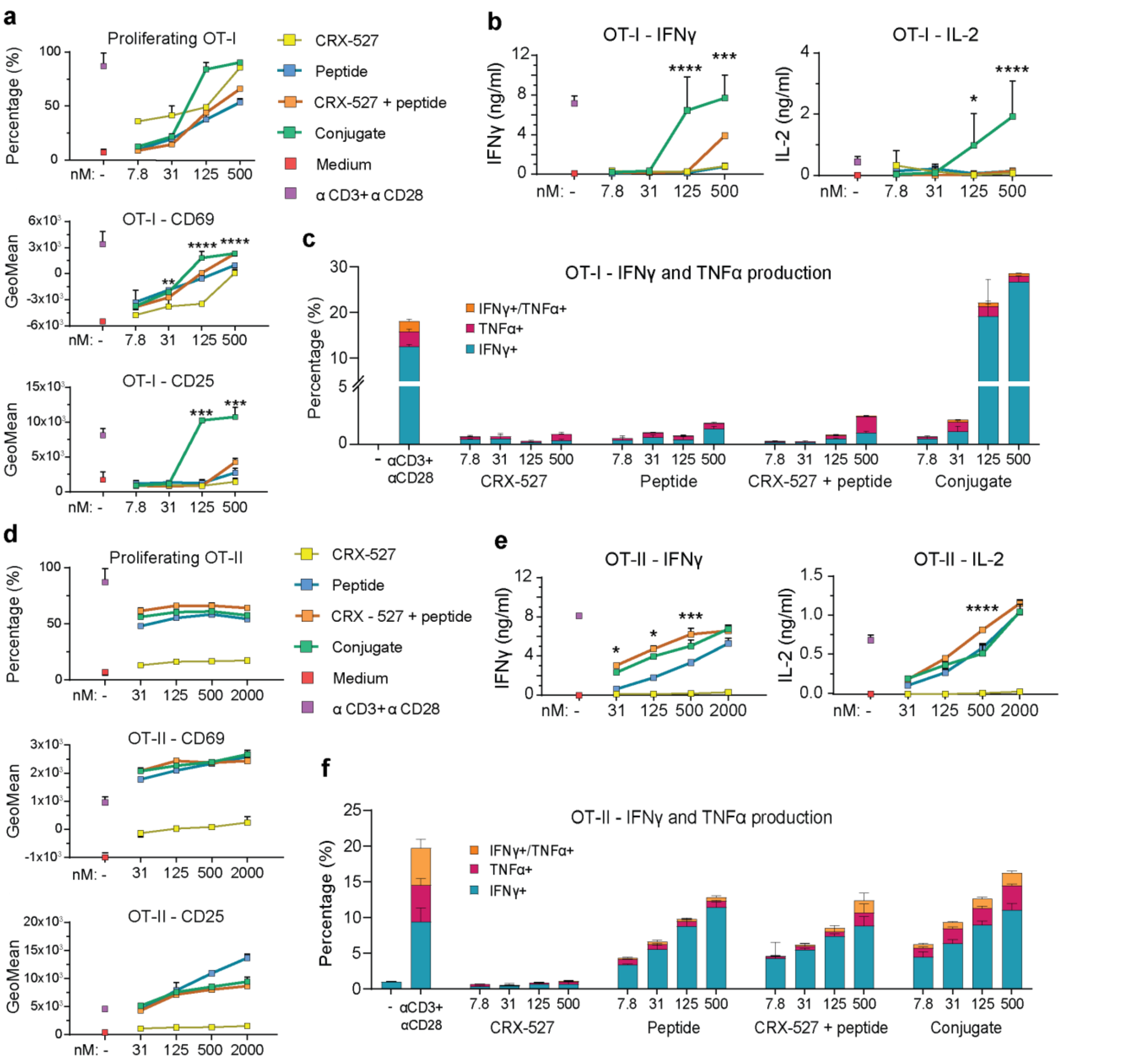

Supplementary Figure 1: CRX-527-peptide conjugates induce OT-I and OT-II cell activation *in vitro*. D1 dendritic cells were pulsed for 2 hours with free peptide or conjugate containing OVA CTL (**a-c**) or OVA Help (**d-f**) epitopes and incubated for 48 hours with purified CFSE-labelled OT-I or OT-II T cells. In the last 5 hours, supernatant was collected for cytokine detection by ELISA and cells were incubated with Brefeldin A followed by staining for activation markers and cytokines. (**a**) Percentage of proliferating cells and expression of CD69 and CD25 activation markers on OT-I cells. Medium and stimulation with αCD3 + αCD28 antibodies were used as negative and positive controls. (**b**) Production of IFNγ and IL-2 cytokines by OT-I as detected in the supernatant by ELISA. (**c**) IFNγ and TNFα cytokine production in OT-I as detected by intracellular cytokine staining. (**d**) Percentage of proliferating cells and expression of CD69 and CD25 activation markers on OT-II cells. Medium and stimulation with αCD3 + αCD28 antibodies were used as negative and positive controls. (**e**) Production of IFNγ and IL-2 cytokines by OT-II as detected in the supernatant by ELISA. (**f**) IFNγ and TNFα cytokine production in OT-II as detected by intracellular cytokine staining. Statistical significance in (**a**) of peptide/mix/conjugate versus CRX-527 was determined by two-way ANOVA followed by Tukey's multiple comparison test. Statistical significance in (**b**) and (**e**) of conjugates versus mix was determined by two-way ANOVA followed by Dunnett's multiple comparison test; * $p < 0.05$, ** $p < 0.01$, *** $p < 0.001$, **** $p < 0.0001$. Experiments were performed in triplicates and are representative of two or three independent experiments with similar results.

Figure S2:

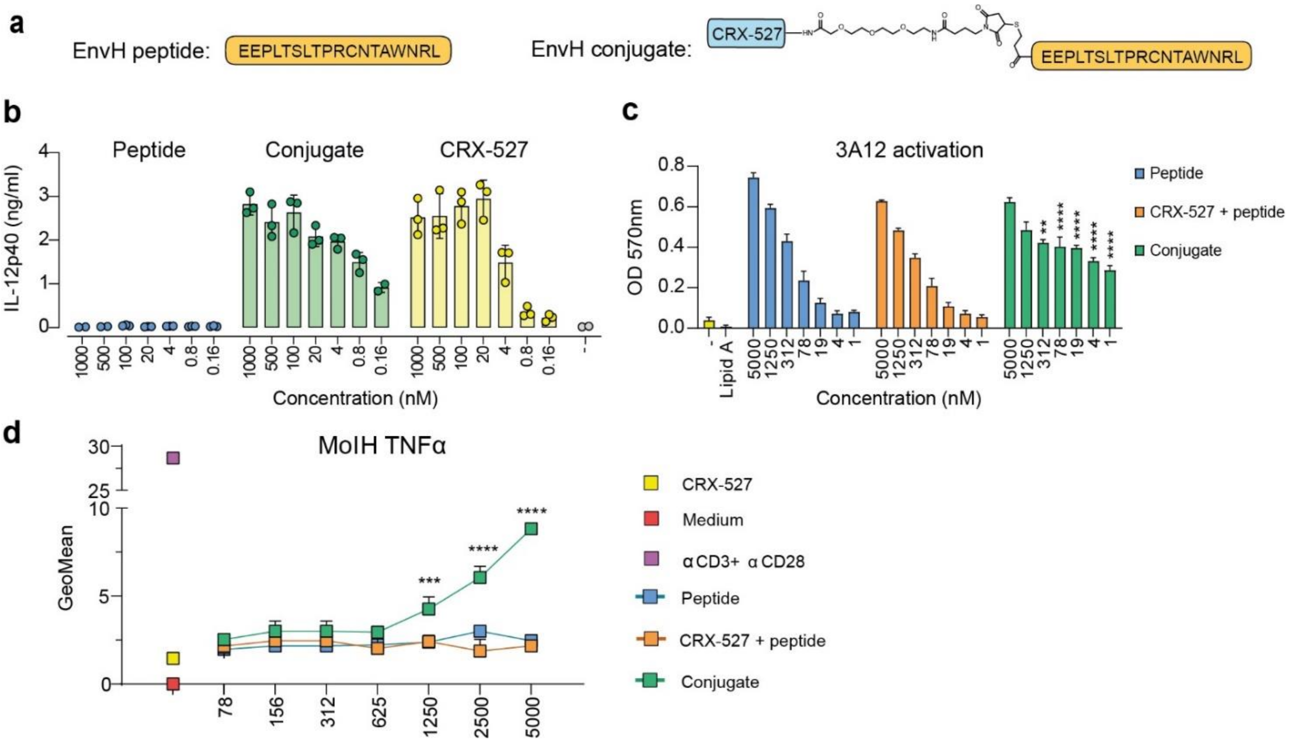

Supplementary Figure 2: CRX-527-envH peptide conjugate matures dendritic cells and enhances CD4 T cell activation. (**a**) Schematic representation of the structures of the Lipid A analogue CRX-527 and the EnvH peptide conjugate. (**b**) Concentration of IL-12p40 in the supernatant of D1 dendritic cells after overnight incubation with the indicated concentrations of EnvH peptide, conjugate, or CRX-527. (**c**) EnvH peptide or conjugate antigen presentation was evaluated by pulsing D1 DCs overnight with the indicated concentration of compounds followed by overnight incubation with Env-specific reporter hybridoma T cell line 3A12. T cell activation was measured by colorimetric reaction (OD570). (**d**) D1 DCs were pulsed overnight with the indicated compounds at different concentrations and incubated with purified naïve MolH CD4 TCR transgenic T cells. Cells were co-incubated with Brefeldin A followed by cytokine staining for TNFα. Statistical significance in all plots of conjugates versus mix was determined by two-way ANOVA followed by Dunnett's multiple comparison test; * p< 0.05, *** p< 0.001, **** p< 0.0001. Experiments were performed in duplicates or triplicates and are representative of two or three independent experiments with similar results.

Figure S3:

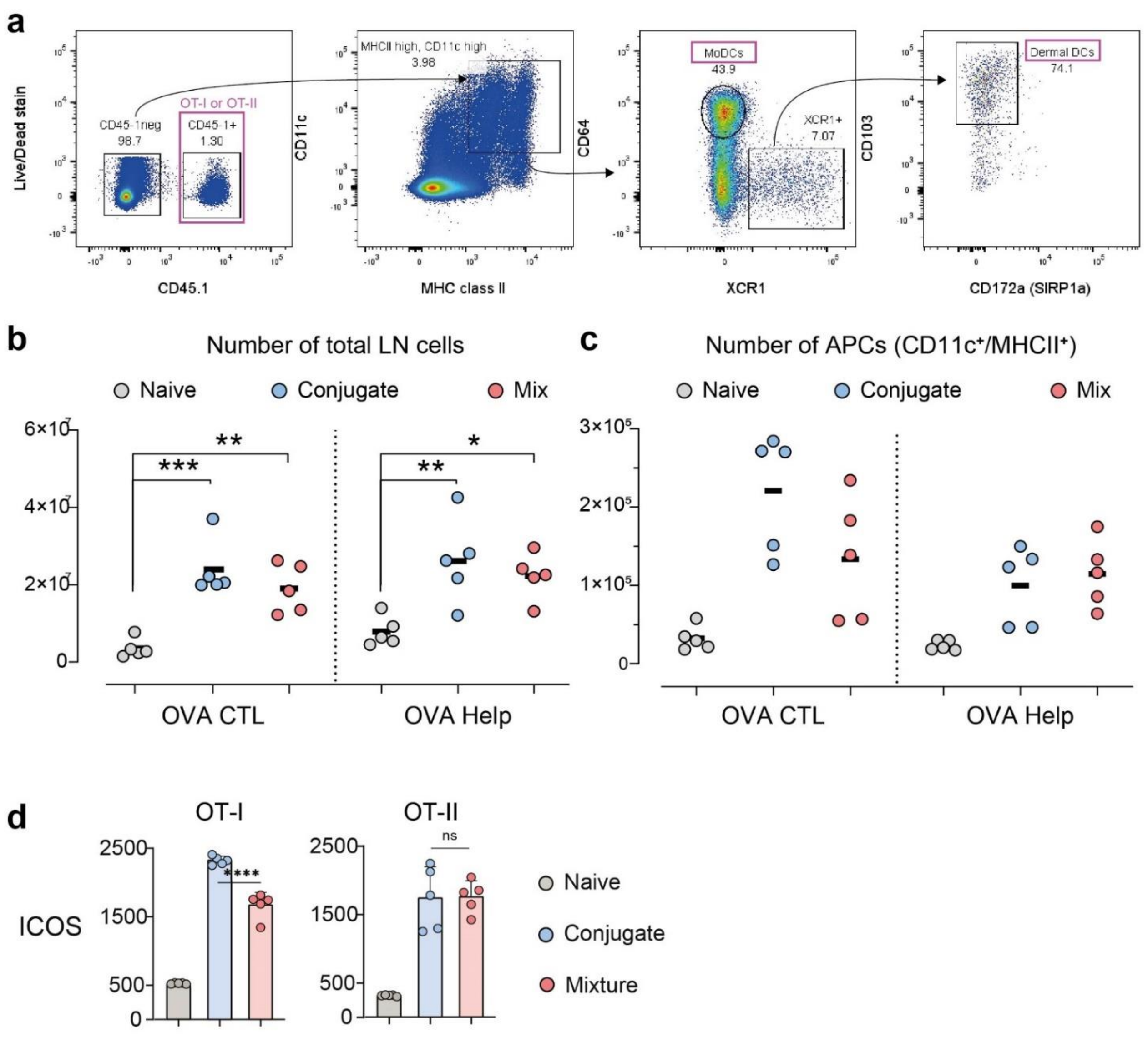

Supplementary Figure 3: OVA CTL and Help CRX-527-peptide conjugates promote APCs influx in the draining lymph node upon *in vivo* injection. Mice (n=5 per group) were adoptively transferred with CFSE labelled OT-I or OT-II 24 hours before receiving 2 nmol of OVA CTL or Help CRX-527conjugates, or an equimolar mix of peptide and CRX-527. 48 hours later the inguinal lymph nodes were harvested for analysis of OT-I or OT-II T cell proliferation and activation. (**a**) Gating strategy for identification of OT-I or OT-II cells (expressing the congenic marker CD45.1) and CD11c+MHCII+ APCs, MoDCs (CD64+XCR1-) and dermal DCs (XCR1+CD103+CD172a-) (**b** and **c**). Absolute count of total cells in the two inguinal lymph nodes (**b**) and APCs (**c**) upon injection of OVA CTL or Help peptides mixed with Lipid A or in form of conjugates. (**d**) Fluorescence intensity of ICOS activation markers as detected by flow cytometry in OT-I or OT-II cells. Statistical significance of the conjugates compared to the mix was determined by one-way ANOVA followed by Sidak’s multiple comparison test; ***** p< 0.0001.

Figure S4:

a

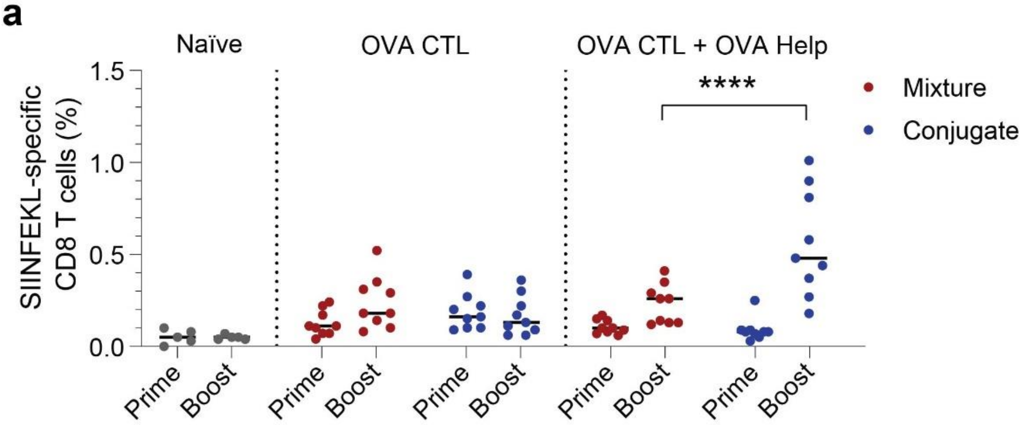

b

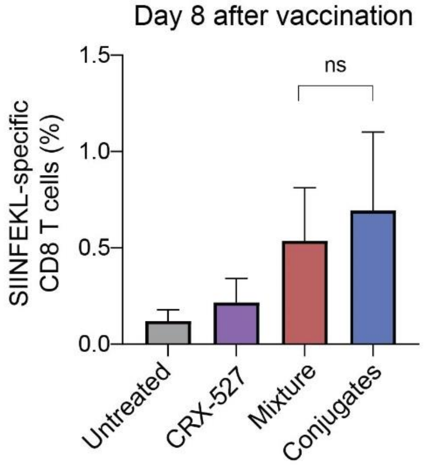

Supplementary Figure 4: OVA CTL and Help CRX-527-peptide conjugates effectively induce SIINFEKL-specific T cell responses upon prophylactic or therapeutic vaccination (**a**) Frequency of SIINFEKL-specific T cells in blood of individual mice (n=5) one week after prime or booster injection with CRX-527 and OVA CTL and Help peptides in conjugated form or mixed before B16OVA tumor challenge. Statistical significance of the conjugates compared to the mix was determined by one-way ANOVA followed by Tukey's multiple comparison test; ***** p< 0.0001. (**b**) Levels of SIINFEKL-specific CD8 T cells detected after therapeutic vaccination in B16OVA tumor-bearing mice. Statistical significance of the conjugates compared to the mix was determined by one-way ANOVA followed by Tukey's multiple comparison test.

Supporting information: Experimental procedure for synthesis of CRX-527-peptide conjugates

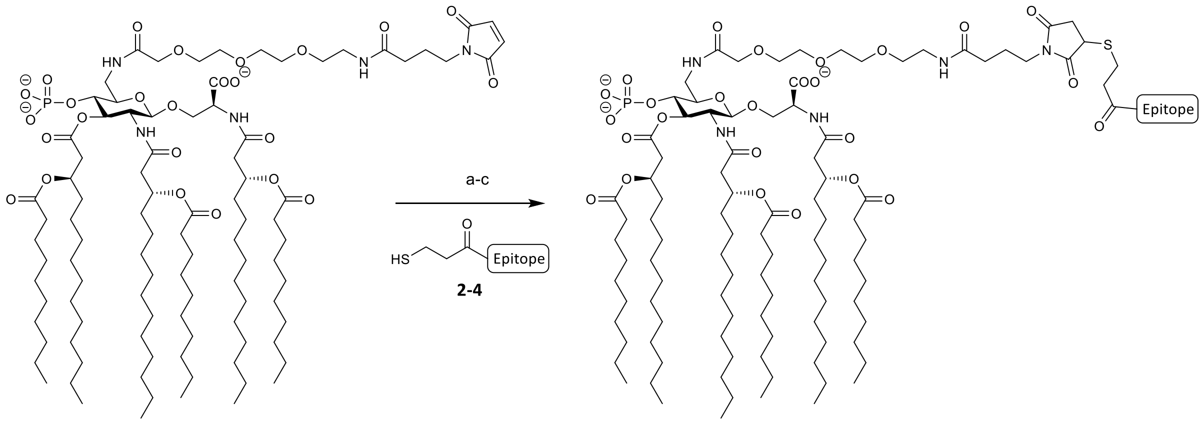

1 **5-7**

Epitope: **5:** HAAHA = ISQAVHAAHAEINEAGRK
6: HPV = GQAEDRAHYNIVTFBBKBDSTLRLBVK
7: EnvH = EEPLTSLTPR-Abu-NTAWNRL

Scheme S1. Synthesis of TLR4-ligand peptide conjugates **5, 6** and **7**. *Reagents and conditions*: a) **2**, DMF/$CHCl_3$/H_2O, 72h, 40%; b) **3**, DMF/$CHCl_3$/H_2O, 72h, 54%. c) **4**, DMF/$CHCl_3$/H_2O, 48h, 38%.

Compound **1**, OVA CTL peptide, OVA CTL conjugate, and OVA Help peptide were synthesized as reported in literature. LC-MS analysis were done on an Agilent Technologies 1260 Infinity system with a C18 Gemini 3 µm, C18, 110 Å, 50 x 4.6 mm column or a Vydac 219TP 5 µm Diphenyl, 150 x 4.6 mm column with a flow of 1, 0.8 or 0.7 ml/min. Absorbance was measured at 214 nm and 256 nm and an Agilent Technologies 6120 Quadrupole mass spectrometer was used as detector. Peptides were purified with a Gilson GX-281 preparative HPLC with a Gemini-NX 5u, C18, 110 Å, 250 x 10.0 mm column or a C6-Phenyl, 110 Å, 250 x 10.0 mm column. Peptide fragments were synthesized with automated solid phase peptide synthesis on an Applied Biosystems 433A Peptide Synthesizer. High resolution mass spectra were recorded on a Synapt G2-Si equipped with an electron spray ion source positive mode. Mass analysis of the TLR4-ligands and TLR4-ligand conjugates was performed on an Ultraflextreme MALDI-TOF or a 15T MALDI-FT-ICR MS system.

Automated solid phase synthesis general experimental information. The automated solid-phase peptide synthesis was performed on a 250 µmol scale on a Protein Technologies Tribute-UV IR Peptide Synthesizer applying Fmoc based protocol starting from Tentagel S RAM resin (loading 0.22 mmol/g). The synthesis was continued with Fmoc-amino acids specific for each peptide. The consecutive steps performed in each cycle for HCTU chemistry on 250 µmol scale: 1) Deprotection of the Fmoc-group with 20% piperidine in DMF for 10 min; 2) DMF wash; 3) Coupling of the appropriate amino acid using a four-fold excess. Generally, the Fmoc amino acid (1.0 mmol) was dissolved in 0.2 M HCTU in DMF (5 mL), the resulting solution was transferred to the reaction vessel followed by 2 mL of 1.0 M DIPEA in DMF to initiate the coupling. The reaction vessel was then shaken for 30 min at 50°C; 4) DMF wash; 5) capping with 10% Ac_2O in 0.1 M DIPEA in DMF; 6) DMF wash; 7) DCM wash. Aliquots of resin of the obtained sequences were checked on an analytical Agilent Technologies 1260 Infinity system with a Gemini 3 µm, C18, 110 Å, 50 x 4.6 mm column or a Vydac 219TP 5 µm Diphenyl, 150 x 4.6 mm column with a 1 ml/min flow. The Fmoc amino acids applied in the synthesis were: Fmoc-Abu-OH, Fmoc-Ala-OH, Fmoc-Asn(Trt)-OH, Fmoc-Asp(O*t*Bu)-OH, Fmoc-Gln(Trt)-OH, Fmoc-Arg(Pbf)-OH, Fmoc-Gln(Trt)-OH, Fmoc-Glu(O*t*Bu)-OH, Fmoc-Gly-OH, Fmoc-His-OH, Fmoc-Ile-OH, Fmoc-Leu-OH, Fmoc-

Lys(Boc)-OH, Fmoc-Lys(MMT)-OH, Fmoc-Phe-OH, Fmoc-Pro-OH, Fmoc-Ser(O*t*Bu)-OH, Fmoc-Thr(O*t*Bu)-OH, Fmoc-Tyr(O*t*Bu)-OH, Fmoc-Val-OH, Fmoc-Val-Thr(psiMe,Mepro)-OH and Fmoc-Asp(O*t*Bu)-Ser(psiMe,Mepro)-OH.

General procedure for cleavage from the resin, deprotection and purification. 30 μmol resin was washed with DMF, DCM and dried after the last synthesis step followed by a treatment for 180 minutes with 0.6 mL cleavage cocktail of 95% TFA, 2.5% TIS and 2.5% H_2O. The suspension was filtered, the resin was washed with 0.6 mL of the cleavage cocktail, and the combined TFA solutions were added dropwise to cold Et_2O and stored at -20°C overnight. The obtained suspension of the product in Et_2O was centrifuged, Et_2O was removed and the precipitant was dissolved in $CH_3CN/H_2O/$*t*BuOH (1/1/1 v/v/v) or DMSO/$CH_3CN/H_2O/$*t*BuOH (3/1/1/1 v/v/v/v). Purification was performed on a Gilson GX-281 preparative RP-HPLC with a Gemini-NX 5u, C18, 110 Å, 250 x 10.0 mm column or a Vydac 219TP 5 μm Diphenyl, 250 x 10 mm column.

MALDI-TOF measurements. MALDI-TOF measurements: 1 μL of a DMSO solution of the compound was spotted on a 384-MTP target plate (Bruker Daltonics, Bremen, Germany) and air-dried. Subsequently 1 μL of 2,5-dihydroxybenzoic acid (2,5-DHB; Bruker Daltonics) matrix (20 mg/mL in ACN/water; 50:50 (v/v)) was applied on the plate and the spots were left to dry prior analysis. Mass analysis of the TLR4-ligand conjugates was performed on a 15T MALDI-FT-ICR MS system.

3-Mercaptoproponamide-Ile-Ser-Gln-Ala-Val-His-Ala-Ala-His-Ala-Glu-Ile-Asn-Glu-Ala-Gly-Arg-Lys-NH_2 (2)

Tentagel S Ram resin loaded with H-Ile-Ser(O*t*Bu)-Gln(Trt)-Ala-Val-His(Trt)-Ala-Ala-His(Trt)-Ala-Glu(O*t*Bu)-Ile-Asn(Trt)-Glu(O*t*Bu)-Ala-Gly-Arg(Pbf)-Lys(MMT)-NH_2 on 70 μmol scale was washed with DMF (5x), followed by the addition of a solution of 3-(tritylthio)propionic acid (51 mg, 150 μmol, 2.1 eq.) and HCTU (58 mg, 140 μmol, 2.0 eq.) in DMF (1.4 mL) and DIPEA (49 μL, 280 μmol, 4.0 eq.). The reaction vessel was shaken overnight at 850 rpm, after which it was washed with DMF (3x) and DCM (3x). The peptide was cleaved from the resin after treatment with TFA/TIS/H_2O (95/2.5/2.5 v/v/v) (2.8 mL) for 3 hours the suspension was filtered and the residue was washed with the cleavage cocktail (2.8 mL). The product was precipitated with Et_2O. After purification by RP-HPLC and lyophilisation, compound **2** (22 mg, 11 μmol, 16%) was obtained as a white solid. LC-MS: Rt = 5.34 min (C18 Gemini, 10 - 50% MeCN, 11 min run); ESI-MS: m/z 995.2 $[M+H]^{2+}$; HRMS: $[M+H]^{2+}$ calcd. for $C_{83}H_{139}N_{29}O_{26}S$: 995.00779, found 995.00816.

3-Mercaptoproponamide-Gly-Gln-Ala-Glu-Pro-Asp-Arg-Ala-His-Tyr-Asn-Ile-Val-Thr-Phe-Abu-Abu-Lys-Abu-Asp-Ser-Thr-Leu-Arg-Leu-Abu-Val-Lys-NH_2 (3)

Tentagel S Ram resin loaded with H-Gly-Gln(Trt)-Ala-Glu(O*t*Bu)-Pro-Asp(O*t*Bu)-Arg(Pbf)-Ala-His(Trt)-Tyr(O*t*Bu)-Asn(Trt)-Ile-Val-Thr(psiMe,Mepro)-Phe-Abu-Abu-Lys(O*t*Bu)-Abu-Asp(O*t*Bu)-Ser(psiMe,Mepro)-Thr(O*t*Bu)-Leu-Arg(Pbf)-Leu-Abu-Val-Lys(MMT) on 70 μmol scale was washed with DMF (5x), followed by the addition of a solution of 3-(tritylthio)propionic acid (50 mg, 150 μmol, 2.1 eq.) and HCTU (58 mg, 140 μmol, 2.0 eq.) in DMF (1.4 mL) and DIPEA (49 μL, 280 μmol, 4.0 eq.). The reaction vessel was shaken overnight at 850 rpm, after which it was washed with DMF (3x) and DCM (3x). The peptide was cleaved from the resin after treatment with TFA/TIS/H_2O (95/2.5/2.5 v/v/v) (2.8 mL) for 3 hours the suspension was filtered and the residue was washed with the cleavage cocktail (2.8 mL). The product was precipitated with Et_2O. After purification by

RP-HPLC and lyophilisation, compound **3** (7.5 mg, 2.4 µmol, 3%) was obtained as a white solid. LC-MS: Rt = 5.00 min (C18 Gemini, 10 - 90% MeCN, 11 min run); ESI-MS: m/z 1594.2 $[M+H]^{2+}$; HRMS: $[M+H]^{5+}$ calcd. for $C_{141}H_{234}N_{41}O_{41}S$: 637.94358, found 637.94327.

3-Mercaptoproponamide-Glu-Glu-Pro-Leu-Thr-Ser-Leu-Thr-Pro-Arg-Abu-Asn-Thr-Ala-Trp-Asn-Arg-Leu-NH_2 (4)

Tentagel S Ram resin was loaded with H-Glu(O*t*Bu)-Glu(OtBu)-Leu-Thr(psiMe,Mepro)-Ser(O*t*Bu)-Leu-Thr(psiMe,Mepro)-Pro-Arg(Pbf)-Abu-Asn(Trt)-Thr(psiMe,Mepro)-Ala-Trp-Asn(Trt)-Arg(Pbf)-Leu-NH_2 on 50 µmol scale was washed with DMF (5x), followed by the addition of a solution of 3-(tritylthio)propionic acid (50 mg, 150 µmol, 2.1 eq.) and HCTU (58 mg, 140 µmol, 2.0 eq.) in DMF (1.4 mL) and DIPEA (49 µL, 280 µmol, 4.0 eq.). The reaction vessel was shaken overnight at 850 rpm, after which it was washed with DMF (3x) and DCM (3x). The peptide was cleaved from the resin after treatment with TFA/TIS/H_2O (95/2.5/2.5 v/v/v) (2.8 mL) for 3 hours the suspension was filtered and the residue was washed with the cleavage cocktail (2.8 mL). The product was precipitated with Et_2O. After purification by RP-HPLC and lyophilisation, compound **4** (4.4 mg, 2.0 µmol, 4%) was obtained as a white solid. LC-MS: Rt = 6.36 min (C18 Gemini, 10 - 90% MeCN, 11 min run).

OVA Help conjugate (5)

Thiol-peptide **2** (2.71 mg, 1.36 µmol, 1.5 eq.) was dissolved in a mixture of DMF/MilliQ H_2O (4/0.5 v/v, 272 µl) in an Eppendorf tube. A solution of compound **1** (5.0 mM, 182 µL, 0.91 µmol, 1.0 eq.) was added and the mixture was shaken at 850 rpm for 48 hours. LCMS analysis showed complete conversion of the starting material. The reaction mixture was diluted with a mixture of CH_3CN/*t*BuOH/MilliQ H_2O (1/1/1 v/v/v, 0.55 mL) and sonicated for 5 minutes. A C18 column was washed subsequently with CH_3CN, MeOH, DCM/MeOH (1/1 v/v), MeOH, CH_3CN, CH_3CN/H_2O + 0.1% TFA, H_2O + 0.1% TFA. The reaction mixture was added on the column and the Eppendorf was rinsed with a mixture of CH_3CN/*t*BuOH/MilliQ H_2O (1/1/1 v/v/v, 0.50 mL), which was also added on the C18 column. The column was subsequently flushed with 6 mL of the follow solvent systems: H_2O + 0.1% TFA, CH_3CN/H_2O + 0.1% TFA (1/1 v/v), CH_3CN, DMSO, CH_3CN/*t*BuOH/MilliQ H_2O (1/1/1 v/v/v) and collected in Eppendorfs containing 1.0 mL of each solvent system. The column was then flush with MeOH (6.0 mL), followed by DCM/MeOH (1/1 v/v, 6.0 mL), which were collected in separate flasks, concentrated *in vacuo* at 35°C and lyophilized by dissolving in CH_3CN/*t*BuOH/MilliQ H_2O (1/1/1 v/v/v), yielding the conjugate as a white solid (1.4 mg, 0.36 µmol, 40%). LC-MS: Rt = 15.95 min (Diphenyl Vydac, 10 - 90% IPA, 25 min run, 0.5 mL/min). ESI-MS: m/z 1916.4 $[M+H]^{2+}$. MALDI-FT-ICR MS (m/z): $[M+H]^{+}$ calcd. for $C_{108}H_{312}N_{34}O_{51}SP$: 3829.2319, found 3829.1598.

HPV conjugate (6)

Thiol-peptide **3** (5.79 mg, 1.82 µmol, 2.0 eq.) was dissolved in a mixture of DMF/MilliQ H_2O (4/0.5 v/v, 273 µl) in an Eppendorf tube. A solution of compound **1** (5.0 mM, 180 µL, 0.91 µmol, 1.0 eq.) was added and the mixture was shaken at 850 rpm for 3 days. LC-MS analysis showed complete conversion of the starting material. The reaction mixture was diluted with a mixture of DMSO (0.55 mL) and sonicated for 5 minutes. After purification by RP-HPLC (Gemini, C6-phenyl) and lyophilization, conjugate **5** (2.2 mg, 0.49 µmol, 54%) was obtained as a white solid. LC-MS: Rt = 15.09 min (Diphenyl Vydac, 10 - 90% IPA, 25 min run, 0.5 mL/min). ESI-MS: m/z 1676.9 $[M+H]^{3+}$. MALDI-FT-ICR MS (m/z): $[M+H]^{+}$ calcd. for $C_{238}H_{404}N_{46}O_{66}SP$: 5025,9124, found 5025.7939.

EnvH Conjugate (7)

Thiol-peptide **4** (1.42 mg, 0.64 µmol, 1.5 eq.) was dissolved in a mixture of DMF/MilliQ H_2O (4/1 v/v, 86.6 µL) in an Eppendorf tube. A solution of compound **1** (5.0 mM, 85 µL, 0.43 µmol, 1.0 eq.) in $CHCl_3$ was added and the reaction was shaken for 48h at 850 rmp. LCMS analysis showed complete conversion of the starting material. After purification by RP-HPLC (Gemini, C6-phenyl) and lyophilization, conjugate **6** (0.7 mg, 0.18 µmol, 38%) was obtained as a white solid. LC-MS: Rt = 14.44 min (Diphenyl Vydac, 10 - 90% IPA, 25 min run, 0.5 mL/min). HRMS $[M+H]^+$ calcd. for $C_{191}H_{325}N_{32}O_{55}PS$: 2007.16403, found 2007,17118.

LCMS spectra

LCMS spectra of peptide **2**

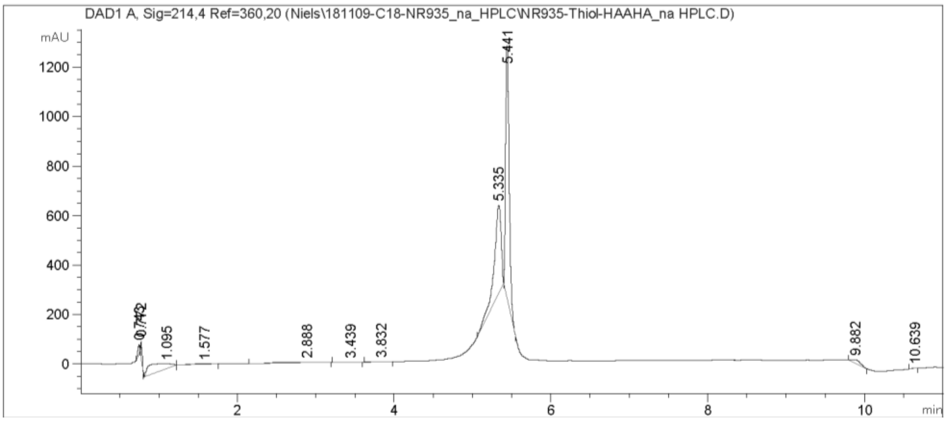

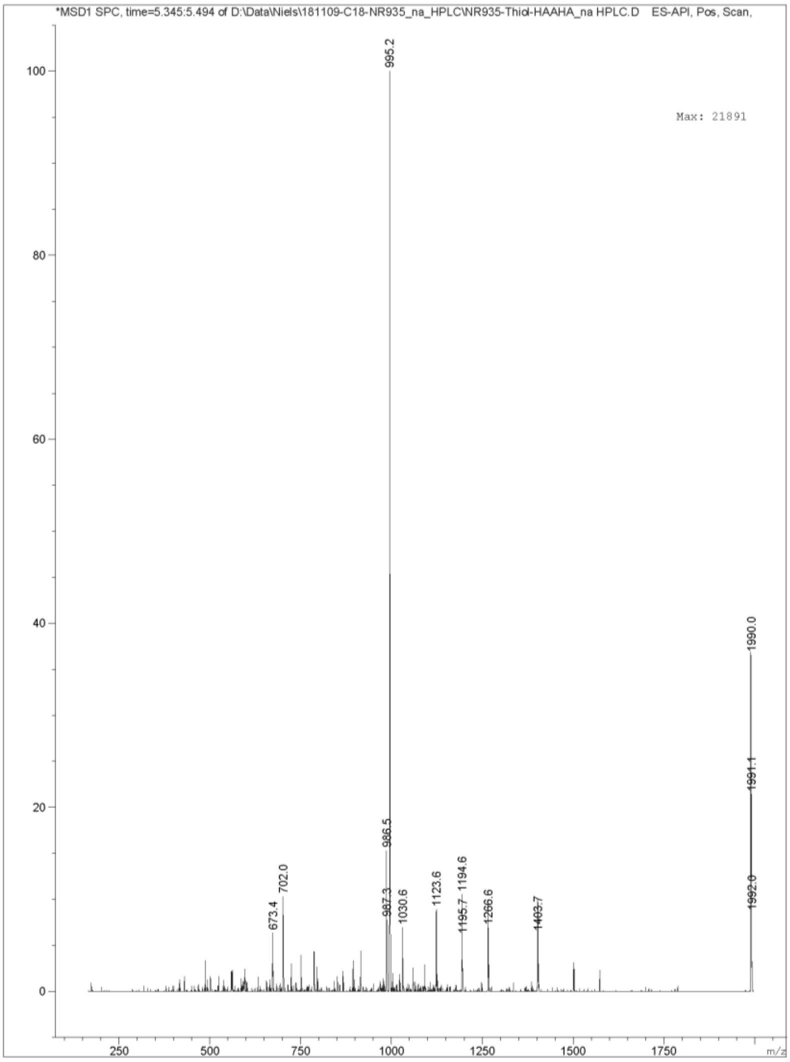

LCMS spectra of peptide **3**

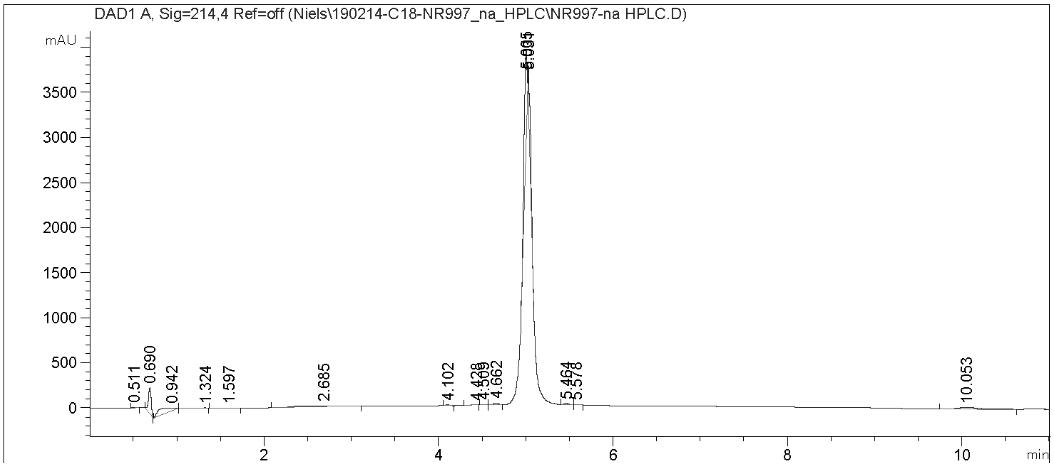

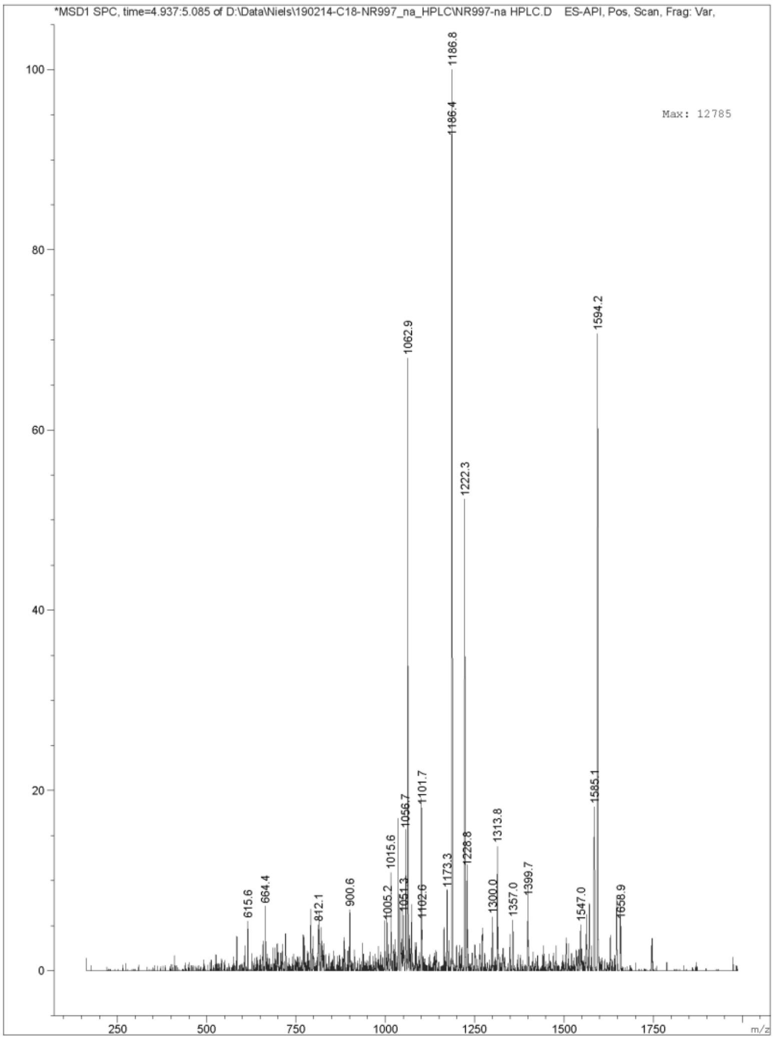

LCMS spectra of peptide **4**

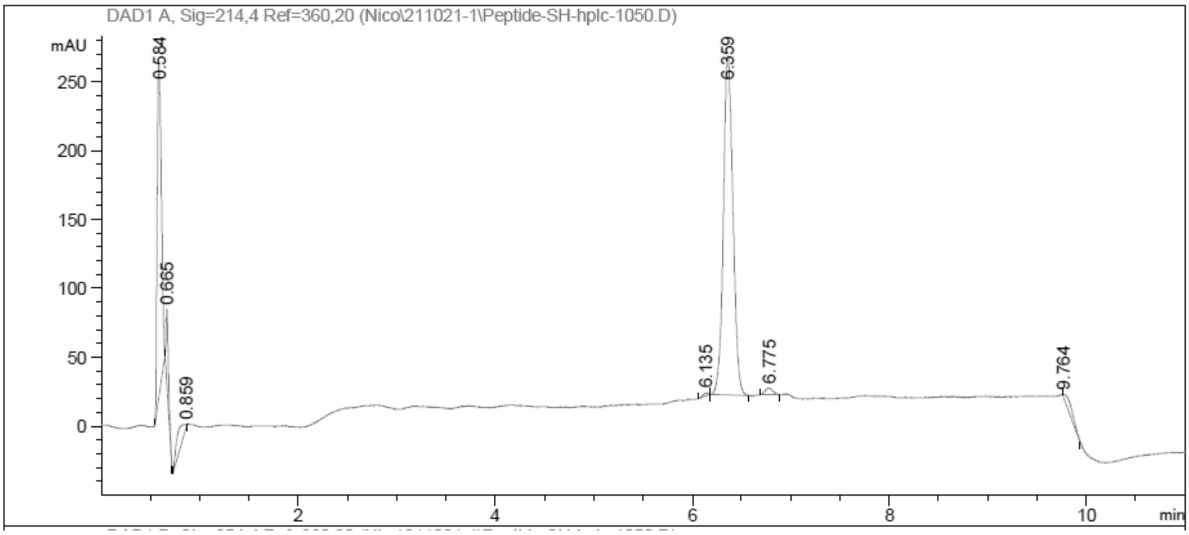

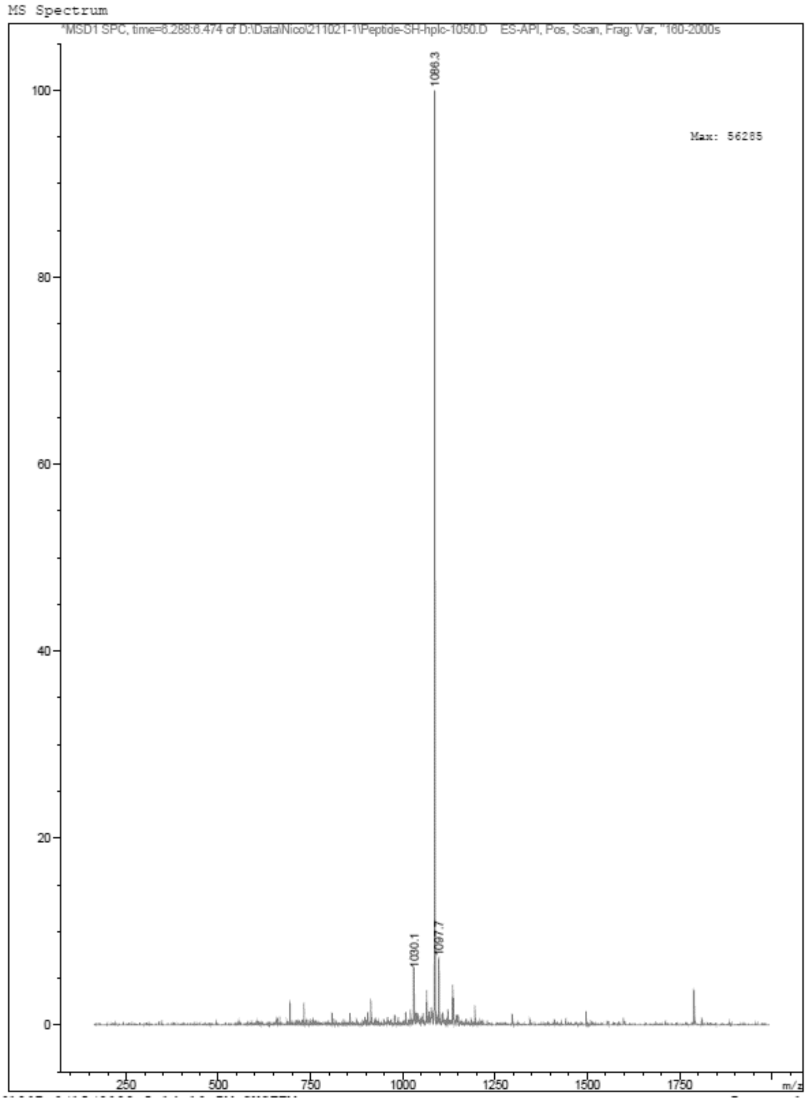

LCMS spectra of OVA Help conjugate **5**

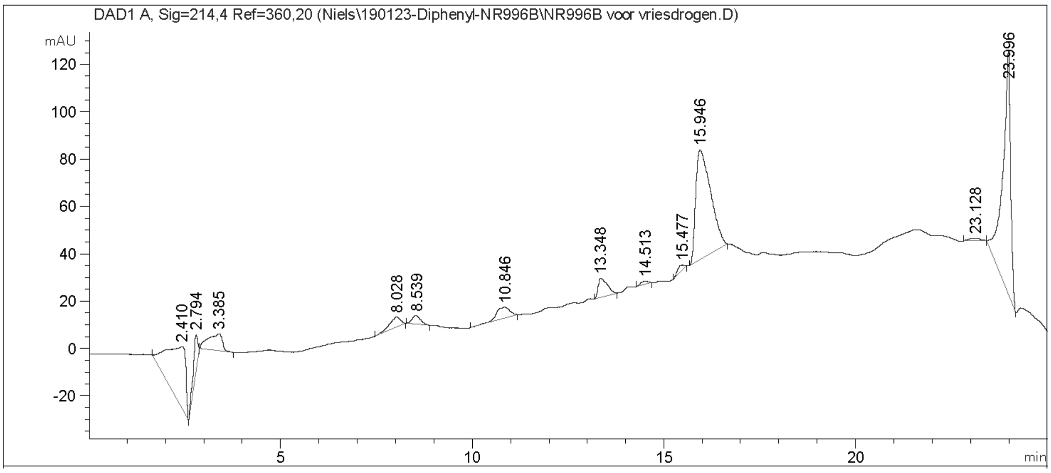

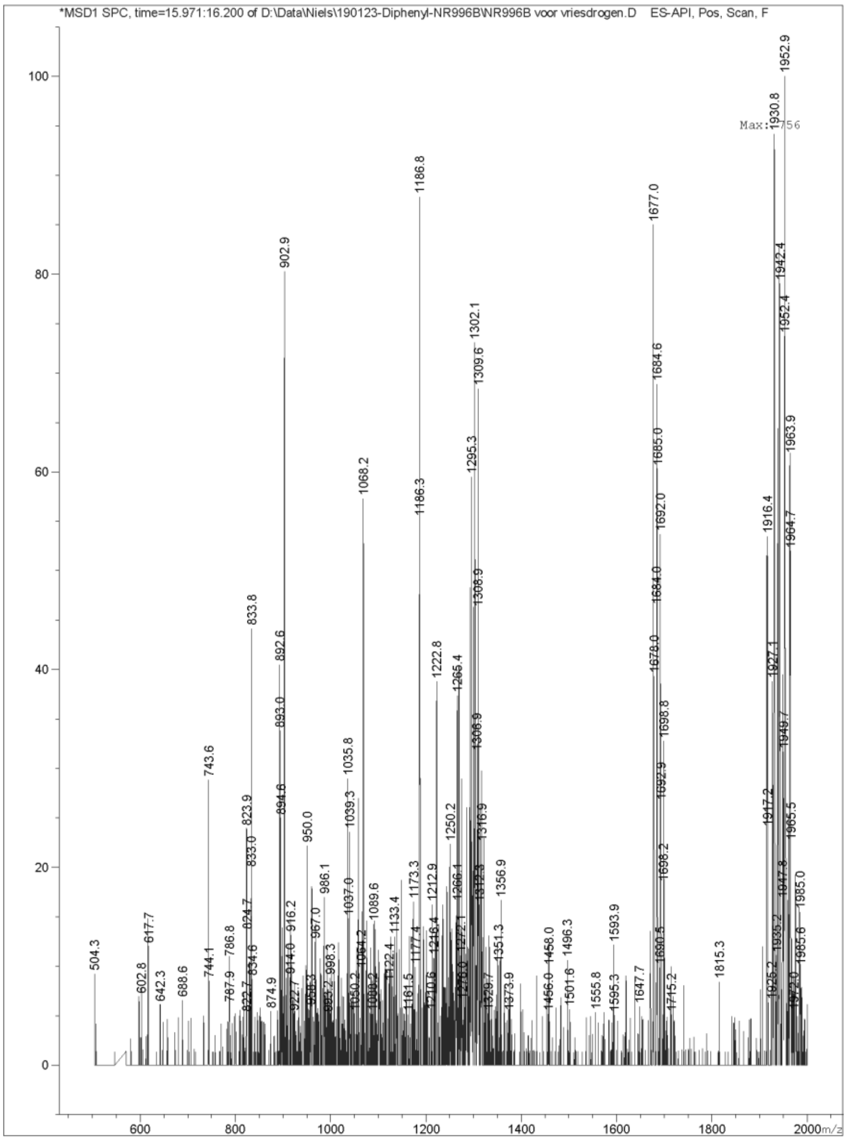

LCMS spectra of HPV conjugate **6**

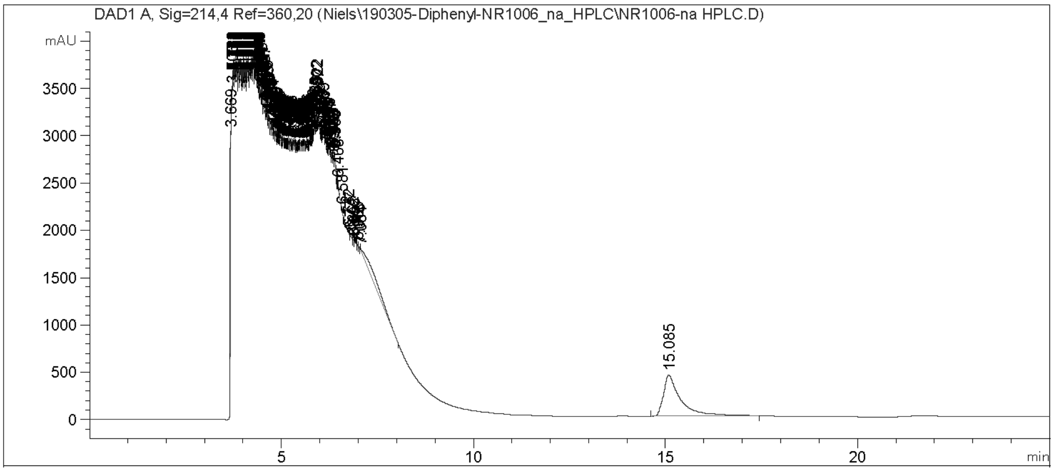

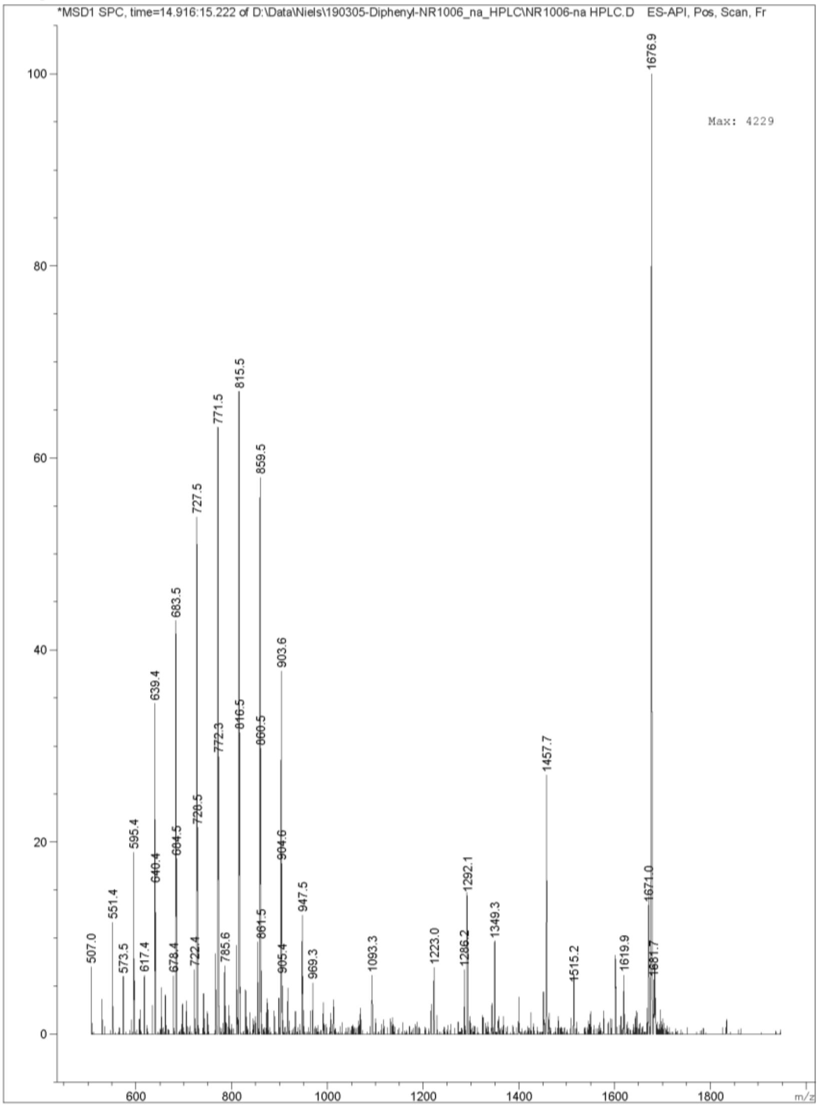

LCMS spectra of EnvH conjugate **7**

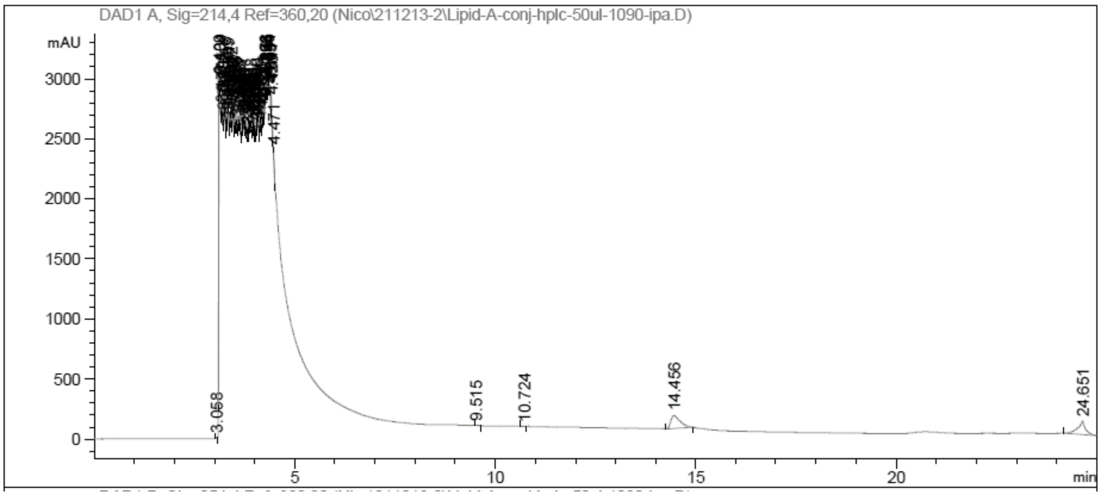

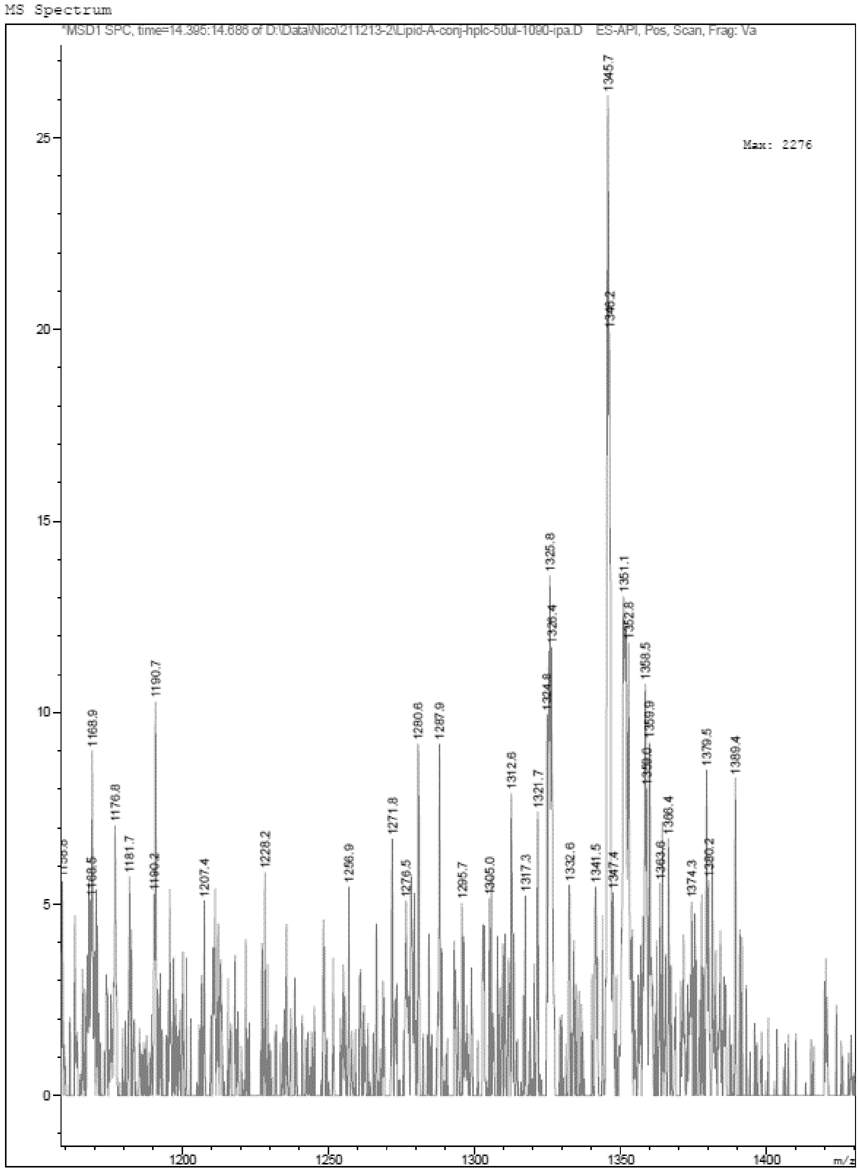

MALDI-FT-ICR mass spectra

OVA Help conjugate **5**

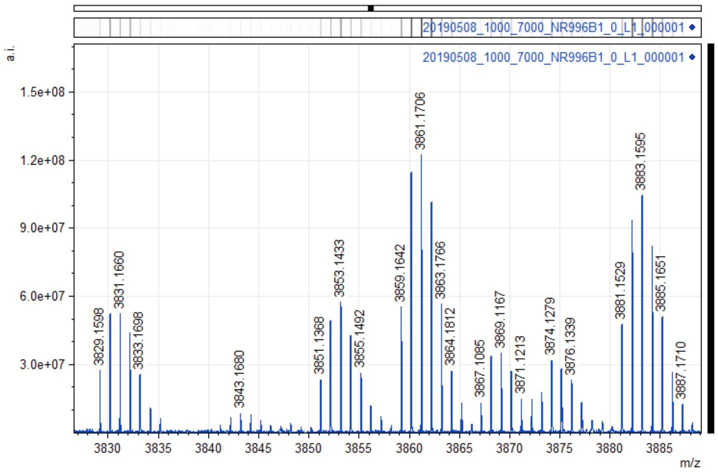

HPV conjugate **6**

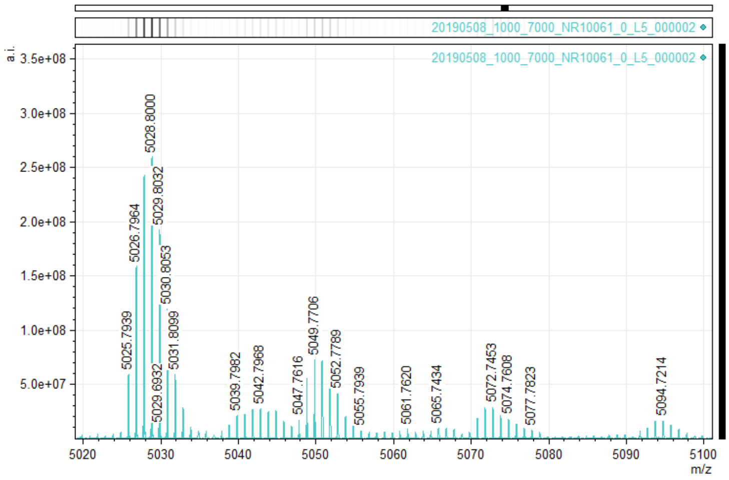

HRMS mass spectrum

EnvH conjugate **7**

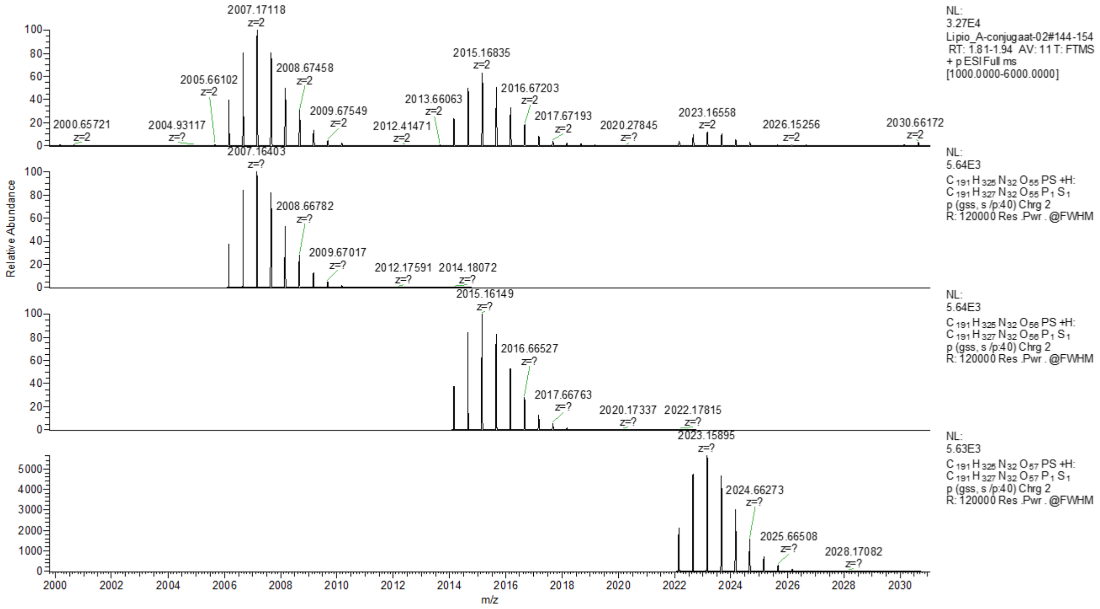

Supplement: Supplementary file 1 — Supplementary Material [file 41541_2022_484_MOESM1_ESM.pdf]
