# Supplementary material for: Lipid A analog CRX-527 conjugated to synthetic peptides enhances vaccination efficacy and tumor control
Source: NPJ Vaccines. 2022 Jun 23;7:64. doi: 10.1038/s41541-022-00484-y (PMC9226002; doi:10.1038/s41541-022-00484-y)
Supplement: Supplementary file 2 — REPORTING SUMMARY [file 41541_2022_484_MOESM2_ESM.pdf]

nature portfolio

Corresponding author(s): Ferry Ossendorp

Last updated by author(s): Feb 23, 2022

Reporting Summary

Nature Portfolio wishes to improve the reproducibility of the work that we publish. This form provides structure for consistency and transparency in reporting. For further information on Nature Portfolio policies, see our Editorial Policies and the Editorial Policy Checklist.

Statistics

For all statistical analyses, confirm that the following items are present in the figure legend, table legend, main text, or Methods section.

n/a	Confirmed	
☐	☒	The exact sample size (*n*) for each experimental group/condition, given as a discrete number and unit of measurement
☐	☒	A statement on whether measurements were taken from distinct samples or whether the same sample was measured repeatedly
☐	☒	The statistical test(s) used AND whether they are one- or two-sided<br>*Only common tests should be described solely by name; describe more complex techniques in the Methods section.*
☒	☐	A description of all covariates tested
☐	☒	A description of any assumptions or corrections, such as tests of normality and adjustment for multiple comparisons
☐	☒	A full description of the statistical parameters including central tendency (e.g. means) or other basic estimates (e.g. regression coefficient) AND variation (e.g. standard deviation) or associated estimates of uncertainty (e.g. confidence intervals)
☐	☒	For null hypothesis testing, the test statistic (e.g. *F*, *t*, *r*) with confidence intervals, effect sizes, degrees of freedom and *P* value noted<br>*Give P values as exact values whenever suitable.*
☒	☐	For Bayesian analysis, information on the choice of priors and Markov chain Monte Carlo settings
☒	☐	For hierarchical and complex designs, identification of the appropriate level for tests and full reporting of outcomes
☐	☒	Estimates of effect sizes (e.g. Cohen's *d*, Pearson's *r*), indicating how they were calculated

Our web collection on statistics for biologists contains articles on many of the points above.

Software and code

Policy information about availability of computer code

Data collection	No code or software for data collection was used.
Data analysis	GraphPad Prism V.8<br>FlowJo

For manuscripts utilizing custom algorithms or software that are central to the research but not yet described in published literature, software must be made available to editors and reviewers. We strongly encourage code deposition in a community repository (e.g. GitHub). See the Nature Portfolio guidelines for submitting code & software for further information.

Data

Policy information about availability of data

All manuscripts must include a data availability statement. This statement should provide the following information, where applicable:

- Accession codes, unique identifiers, or web links for publicly available datasets
- A description of any restrictions on data availability
- For clinical datasets or third party data, please ensure that the statement adheres to our policy

The data generated during the current study are available from corresponding author upon reasonable request.

Field-specific reporting

Please select the one below that is the best fit for your research. If you are not sure, read the appropriate sections before making your selection.

☒ Life sciences ☐ Behavioural & social sciences ☐ Ecological, evolutionary & environmental sciences

For a reference copy of the document with all sections, see nature.com/documents/nr-reporting-summary-flat.pdf

Life sciences study design

All studies must disclose on these points even when the disclosure is negative.

Sample size	For T cell proliferation experiment, a group size of 5 mice was used. This was based on previous experiments done in the past years, which do not display high variations and a group size of 5 mice is sufficient to provide statistical significance.<br>For tumor experiments, group size of sizes were calculated using the sample size calculator from http://biostat.mc.vanderbilt.edu/wiki/Main/PowerSampleSize, selecting the "sample size" option for survival and "hazard ratio or relative risk" option. Input paramaters are based upon previous experiments with B16-OVA tumors. Alpha: 0.05, Power: 0.8 R: 0.25, M1: 21 days , A: 0, F: 60 days and a control to experimental group (M) of 1. Resulting sample size is 9 mice per group. In some experiments, 10 mice were included to cover the risk of low tumor take.
Data exclusions	No data were excluded from analysis.
Replication	All n vitro experiments were executed in triplicates and repeated 2 or 3 times. For in vivo experiments, sample size was chosen to ensure statistical significance.
Randomization	All animals were randomly assigned for vaccination groups.
Blinding	Tumor measurements were taken blindly.

Reporting for specific materials, systems and methods

We require information from authors about some types of materials, experimental systems and methods used in many studies. Here, indicate whether each material, system or method listed is relevant to your study. If you are not sure if a list item applies to your research, read the appropriate section before selecting a response.

Materials & experimental systems

n/a	Involved in the study	
☐	☒	Antibodies
☐	☒	Eukaryotic cell lines
☒	☐	Palaeontology and archaeology
☐	☒	Animals and other organisms
☒	☐	Human research participants
☒	☐	Clinical data
☒	☐	Dual use research of concern

Methods

n/a	Involved in the study	
☒	☐	ChIP-seq
☐	☒	Flow cytometry
☒	☐	MRI-based neuroimaging

Antibodies

Antibodies used

ELISA antibody pairs were purchased from BioLegend :
- purified anti-mouse IFNg (BioLegend, 505702)
- Biotin anti-mous IFNg (BioLegend, 505804)
- purified anti-mouse IL-2 (BioLegend, 503702)
- Biotin anti-mouse IL-2 (BioLegend, 503804)
-HRP streptavidin (BioLegend =, 405210)

For Flow cytometry antibody used were:
- Alexa Fluor® 700 anti-mouse CD45.1 (BioLegend, 110723)
- Brilliant Violet 650™ anti-mouse CD19 (BioLegend, 115541)
- Brilliant Violet 711™ anti-mouse CD64 (BioLegend, 139311)
- Brilliant Violet 785™ anti-mouse/rat XCR1 (BioLegend, 148225)
- Brilliant Violet 605™ anti-mouse Ly-6C (BioLegend, 128035)
- Brilliant Violet 711™ anti-mouse CD25 (BioLegend, 102049)
- PerCP/Cyanine5.5 anti-mouse CD172a (SIRPα) (BioLegend, 144009)
- PE/Dazzle™ 594 anti-mouse TNF-α (BioLegend, 506345)
- PerCP/Cyanine5.5 anti-mouse CD8a (BioLegend, 100733)
- Alexa Fluor® 700 anti-mouse CD4 (eBioscience, 56-0041-82)
- V500 Rat Anti-Mouse I-A/I-E (BD Biosciences, 562366)
- APC-R700 Rat Anti-Mouse CD8a (BD Biosciences, 564983)

- PE-Cy™7 Rat Anti-Mouse IFN-γ (BD Biosciences, 557649)
- PE-Cy™7 Rat Anti-Mouse CD86 (BD Biosciences, 560582)
- eFluor 450 anti-mouse CD45.1 (eBioscience, 48-0453-82)
- PE anti-mouse CD103 (eBioscience, 12-1031-82)
- Fixable Viability Dye eFluor™ 780 (eBioscience, 65-0865-14)

Validation: Commercial antibodies were used according to manufacturer instructions.

Eukaryotic cell lines

Policy information about cell lines

Cell line source(s)	D1 cell line were a gift from P. Ricciardi-Castagnoli.<br>the B3Z, OTIIZ and A3Z hybridoma were produced in our laboratory.<br>B16-OVA and TC-1 were present in our laboratory.
Authentication	The hybridoma cell lines were established by clonal selection and authenticated for recognition of the peptide against which they were raised. The other cell lines were authenticated by the people who provided them.
Mycoplasma contamination	Cell lines are regularly tested fro mycoplasma contamination.
Commonly misidentified lines (See ICLAC register)	No misidentified cell lines are present in this study.

Animals and other organisms

Policy information about studies involving animals; ARRIVE guidelines recommended for reporting animal research

Laboratory animals	6-8 weeks old C57BL/6 females were used for all in vivo studies. The TCR transgenic OT-I and OT-II mouse strains were obtained from Jackson Laboratory and maintained on CD45.1+ C57BL/6 background. The TCR transgenic MolH strain was created and bred in-house. For TCR transgenic mouse experiments, females below 1 year of age were used.
Wild animals	n.a.
Field-collected samples	n.a.
Ethics oversight	All animal experimentations were approved by and according to guidelines of the Dutch Animal Ethical Committee.

Note that full information on the approval of the study protocol must also be provided in the manuscript.

Flow Cytometry

Plots

Confirm that:

☐ The axis labels state the marker and fluorochrome used (e.g. CD4-FITC).

☒ The axis scales are clearly visible. Include numbers along axes only for bottom left plot of group (a 'group' is an analysis of identical markers).

☒ All plots are contour plots with outliers or pseudocolor plots.

☐ A numerical value for number of cells or percentage (with statistics) is provided.

Methodology

Sample preparation	Cells from spleens or lymph nodes were incubated with Liberase/DNase and passed through a cell strainer. In vitro cultures or blood samples were transferred to the staining plate. Erythrolysis was performed and cells were washed with PBA (0.5% BSA and 0.2% NaN2 in PBS). Staining was performed with antibodies in PBA for 30 min on ice. For intracellular staining, cells were fixed in 1% PFA for 20 min at room temperature and stained with antibodies in Permwash solution.
Instrument	BD LSR II
Software	FlowJo
Cell population abundance	for in vitro experiments, 50.000 cells/sample were acquired. For lymph node analysis, around 2 million cells per mouse were acquired. For tetramer staining in blood, a number between 100.000 and 200.000 cells were acquired.
Gating strategy	Gatng strategy for ex vivo lymph node DCs and OT I is shown in supplementary figures.<br>For in vitro readount, TCR transgenic cells were identified by gating on viable cells positive for the CD45.1 congenic marker.<br>For tetramer staining in blood, tetramers-positive cells were identified in CD3+CD8+ positive cells after live/dead gate.

☒ Tick this box to confirm that a figure exemplifying the gating strategy is provided in the Supplementary Information.
